# Highly Regioselective Direct C-H Arylation: Facile Construction of Symmetrical Dithienophthalimide-Based *π*-Conjugated Molecules for Optoelectronics

**DOI:** 10.34133/2020/9075697

**Published:** 2020-08-30

**Authors:** Xiang-Chun Li, Yibo Xue, Wan Song, Yu Yan, Jie Min, Fang Liu, Xu Liu, Wen-Yong Lai, Wei Huang

**Affiliations:** ^1^Key Laboratory for Organic Electronics and Information Displays, Institute of Advanced Materials (IAM), Nanjing University of Posts & Telecommunications, 9 Wenyuan Road, Nanjing 210023, China; ^2^Frontiers Science Center for Flexible Electronics, Xi'an Institute of Flexible Electronics (IFE) and Xi'an Institute of Biomedical Materials & Engineering, Northwestern Polytechnical University, 127 West Youyi Road, Xi'an 710072, China

## Abstract

Controllable direct C-H arylation with high regioselectivity is highly desirable yet remains a formidable challenge. Herein, a facile regioselective direct C-H arylation is developed for efficient construction of a variety of symmetrical dithienophthalimide-based *π*-conjugated molecules. The resulting methodology is applicable to a wide range of substrates, from electron-rich units to electron-deficient units with large steric end groups. Aryl halides have been confirmed to be able to couple with dithienophthalimide (DTI) via direct C-H arylation, showing high regioselectivity. Varying the functional end groups onto the DTI core has been demonstrated to fine tune the emission colors to cover most of the visible spectra. The results suggest a facile strategy towards highly selective direct C-H arylation, opening the prospects towards efficient construction of *π*-conjugated molecules for various potential optoelectronic applications.

## 1. Introduction

**Scheme 1:**
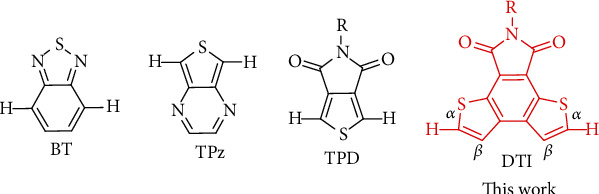
Chemical structures of benzothiadiazoles (BT), thienopyrazines (TPz), thienopyrrolediones (TPD), and dithienophthalimides (DTI).

Organic *π*-conjugated molecules have been intensively explored over recent years for various potential applications, i.e., as active materials for organic light-emitting diodes (OLEDs) [1–7], organic photovoltaics [8–10], organic field-effect transistors [11–14], organic semiconductor lasers [15–19], theranostics [20–22], etc. The construction of organic conjugated molecules depends on the efficient formation of the carbon-carbon bond between two sp^2^ carbons [23–27]. For this purpose, a variety of carbon-carbon cross-coupling reactions, such as Suzuki, Stille, Heck, and Kumada reactions, have thus been widely explored for the couplings of aryl halides with the toxic intermediates (organometallic/boride heteroaromatics) [28–33]. In contrast, direct C-H arylation overcomes the use of toxic intermediates and simplifies the reaction process, which has emerged most recently as a simple, atom-efficient, and ecofriendly methodology for constructing conjugated molecules, especially for those containing electron-deficient moieties [34–39]. Due to the intrinsic electron affinity of electron-deficient moieties, it is generally quite challenging to obtain suitable active intermediates for Suzuki or Stille cross-coupling reactions, since the resulting nucleophilic addition of electron-deficient acceptors would often cause subsequent decomposition [40–43]. Direct C-H arylation offers an effective methodology for the functionalization of electron-deficient moieties without electrophilic substitution or strongly nucleophilic intermediates. To date, only quite a limited number of electron-deficient moieties have been reported to be suitable for direct C-H arylation such as benzothiadiazoles (BT) [44], thienopyrazines (TPz) [45], and thienopyrrolediones (TPD) (Scheme 1) [46]. In order to construct diverse organic *π*-conjugated molecules as efficient active materials for various potential applications, the screening and development of novel building blocks especially those with electron-deficient moieties suitable for direct C-H arylation are preferred but challenging.

For direct C-H arylation, the regioselectivity is a critical issue since the diverse C-H groups may lead to the formation of a variety of isomers and structural defects [47–50]. The isomers and structural defects may increase the difficulty of separating the mixture of the products, which would induce structural uncertainty and may deteriorate the physical properties [51–53]. When considering cross-coupling reactions, it should be noted that structural defects, i.e., isomerization or branching, cannot be eliminated by subsequent purification because they are usually chemically embedded in the chemical structures, which would normally limit the physical performance for optoelectronic applications mainly due to the chemical impurity. Even a small number of structural defects could result in a sharp decline in the optoelectronic characteristics [54–56]. For these reasons mentioned above, the selectivity and regioselectivity issues have been considered the Achilles heel of C-H arylation. Although a variety of direct C-H arylation has been explored [34–39, 44–46], the selective direct C-H arylation reactions are still relatively rare [50, 57–59]. Controllable direct C-H arylation with high selectivity and regioselectivity is thus highly desirable yet remains a formidable challenge.

**Scheme 2:**
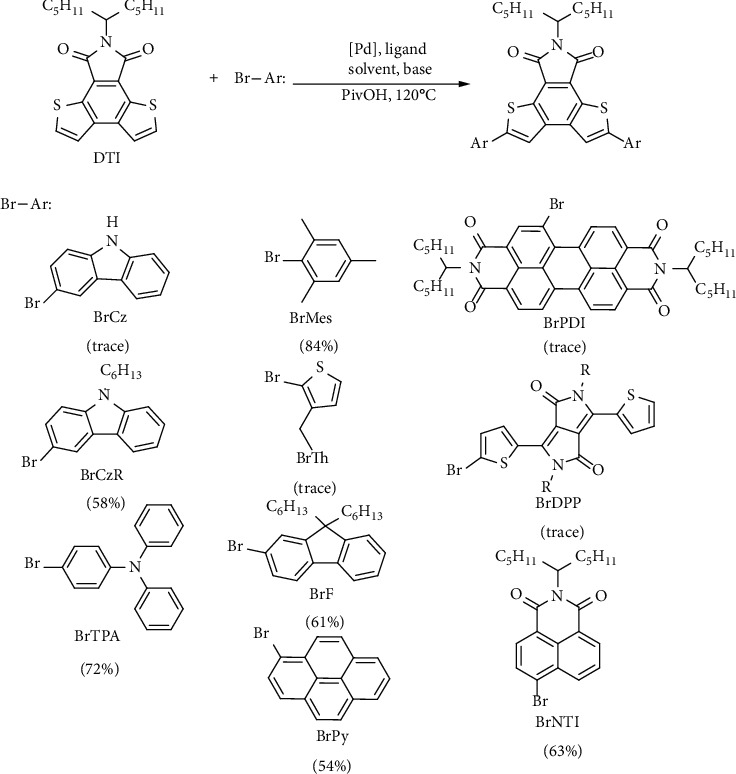
Synthetic routes toward conjugated materials based on DTI by direct C-H arylation with various aryl brominated substituents.

Here, we report a highly regioselective direct C-H arylation based on a new fused-ring electron-deficient dithienophthalimide (DTI) building block and afford a series of symmetrical DTI-based conjugated molecules (Scheme 2). This work includes the optimization of reaction parameters and the adjustment of emission spectra of the *π*-conjugated materials in the solid films with a broad scope ranging from 400 nm to 780 nm. The aromatic halides were successfully converted into target diarylated DTI derivatives with high yields. The general potential of this reaction has been studied by using several aryl substituents such as electron-rich groups (fluorene, pyrene, carbazole and triphenylamine), electron-deficient groups (naphthalene diimide, perylene diimide), and large steric groups (mesitylene) (Scheme 2). Interestingly, direct C-H arylation based on DTI shows excellent regioselectivity as compared with traditional Suzuki or Stille cross-coupling routes. All the DTI-based *π*-conjugated molecules are easy to synthesize and functionalize in a practical, high yield, and selective synthetic pathway, which is beneficial for establishing a library of organic optoelectronic materials with emission wavelengths covering most of the visible spectra. The high isolated yields of diarylated products suggest that C-H activation is feasible and effective to construct DTI-based conjugated molecules. Furthermore, DTI2F and DTI2CzR based on DTI with fluorene and carbazole functional end groups exhibit high fluorescence quantum efficiency of 59% and 67%, respectively. Preliminary OLEDs based on DTI2F demonstrated promising device characteristics with the maximum current efficiency of 3.8% and the maximum brightness of 7200 cd/m^2^ at 11 V. The results suggest a facile strategy towards highly selective direct C-H arylation for the efficient construction of various *π*-conjugated molecules based on DTI for optoelectronic applications.

## 2. Results

### 2.1. Synthetic Optimization

Scheme S1 depicts the synthesis route of DTI. Optimization of reaction conditions was carried out by using DTI and bromomesitylene (BrMes) as C-H arylation substrates. The ligands in tetrahydrofuran (THF) were prescreened before the synthetic study (Table 1). Among various ligands, P(2-MeOPh)_3_ was proven to be effective to afford the target material DTI2Mes with an isolated yield of 30%. The stronger base Cs_2_CO_3_ resulted in a higher yield (33%, entry 5). When the catalyst Pd(OAc)_2_ changed to Pd_2_(dba)_3_, the yield of DTI2Mes was further raised to 44%. Moreover, under the same conditions, a higher yield of DTI2Mes was obtained with using direct C-H arylation in *o*-xylene (84%, Table 1, entry 9). In each entry of Table 1, only a monosubstituted byproduct was obtained. Excessive BrMes (3 equiv.) was used to inhibit side reactions.

Investigation of the (hetero)aryl bromide substrate was carried out by optimizing the reaction conditions: Pd_2_(dba)_3_ (0.03 equiv.), P(2-MeOPh)_3_ (0.12 equiv.), PivOH (1.0 equiv.), and Cs_2_CO_3_ (3.0 equiv.) in *o*-xylene at 120°C for 24 h. Under the optimized conditions, the substrate (BrMes) with large steric hindrance groups was successfully coupled with DTI to afford product DTI2Mes in high yields (84%). Then, representative electron-rich groups including fluorene, pyrene, carbazole, and triphenylamine were examined to afford donor-acceptor type conjugated molecules in moderate to good isolated yields (58-72%). However, when using electron-rich bromothiophenes as the reaction substrates, only trace target compounds were obtained mainly due to the undesired homocoupling reactions and debromination byproducts of bromothiophenes. The reaction yield of DTI with BrCz was lower than that of its derivative (BrCzR), due to the existence of NH reactive groups in carbazole molecules which are prone to nucleophilic substitution. Subsequently, examination of the substrate scope was extended to electron-deficient (hetero)aryl bromides (BrPDI, BrDPP, and BrNTI). Unfortunately, we failed to isolate pure products DTI2PDI and DTI2DPP because of the quite low yields. DTI2NTI with naphthalimide (NTI) as acceptor-type end groups was successfully obtained at a 63% yield. The favorable isolated yields of different substrates for the diaryl products suggest that direct C-H arylation is an attractive synthetic methodology for the construction of diverse organic *π*-conjugated molecules based on DTI.

### 2.2. Regioselectivity of C-H Arylation Based on DTI

**Scheme 3:**
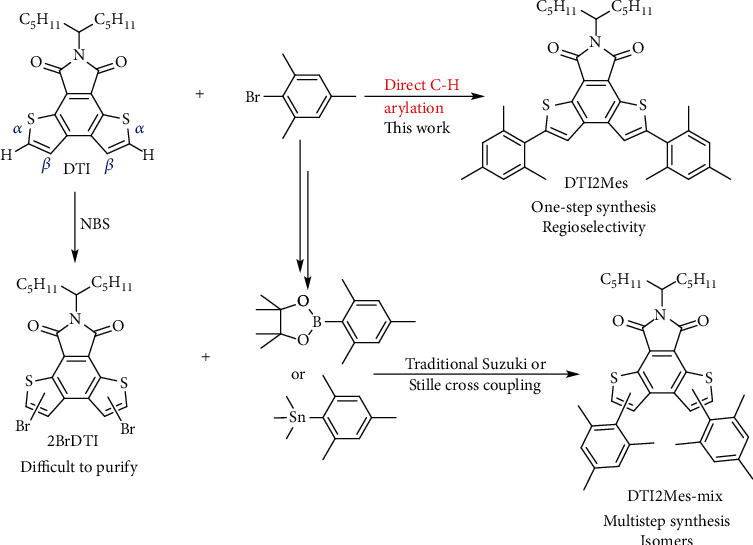
The synthetic routes of DTI2Mes by direct C-H arylation as compared with those of traditional Suzuki-Stille reactions.

**Figure 1:**
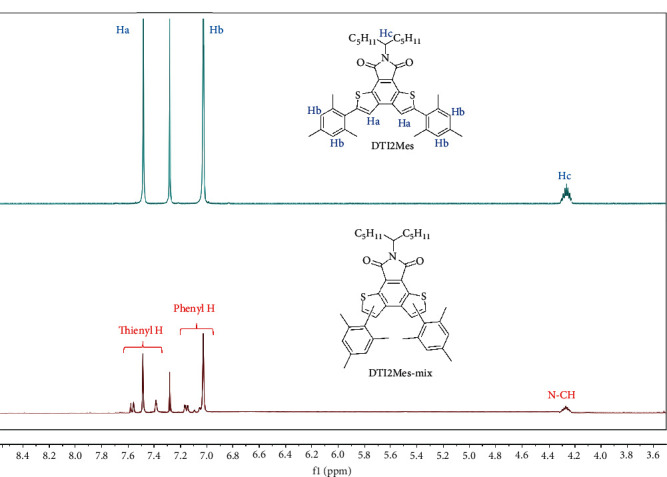
^1^H NMR spectra of DTI2Mes as compared with DTI2Mes-mix.

C-H arylation with poor selectivity is often considered to be a detrimental side reaction in constructing organic *π*-conjugated molecules, which would subsequently limit the optoelectronic performance. Generally, it is difficult for the substrates with multiple C-H bonds to carry out selective C-H arylation, and the reaction conditions are also critical to determine the reactivity of the protons. Surprisingly, the C-H arylation based on DTI exhibits quite high selectivity. Although the reaction was also carried out through traditional Suzuki or Stille cross couplings, it was not applicable to construct selective DTI-based conjugated molecules due to the difficulty in purifying the dibrominated DTI (Scheme 3). As shown in the ^1^H NMR of 2BrDTI (Figure S2), there were three isomers which appeared to be very difficult to isolate separately from the resulting products. In addition, the isolated yield of 2BrDTI was rather low (~10%), since monobromo-, tribromo-, and tetrabromo-substituted DTI byproducts were formed. However, in ^1^H NMR spectra of DTI2Mes synthesized by C-H arylation, all signals were clearly assigned, further corroborating the high regioselectivity of the direct C-H arylation based on DTI (Figure 1). The aromatic region of DTI2Mes (7.03-7.48 ppm) obtained by C-H arylation showed no other signals, while it showed five aromatic protons signals in this region as compared with that of DTI2Mes-mix synthesized by traditional cross couplings. Obviously, there were no other isomers generated during C-H arylation. According to the NMR data, no additional diaryl-substituted isomers were obtained except symmetrical DTI2Mes via C-H arylation. In order to further confirm this, single-crystal X-ray diffraction (XRD) was measured to analyze the structure of DTI2Mes (Table S1). The XRD structure also shows that the reactive site of C-H arylation based on DTI is the *α*-position (Figure S3). Therefore, the direct C-H arylation based on DTI to construct *π*-conjugated organic molecules is not only atom-efficient and ecofriendly but also highly selective, as compared with the traditional Suzuki and Stille cross-coupling routes.

**Scheme 4:**
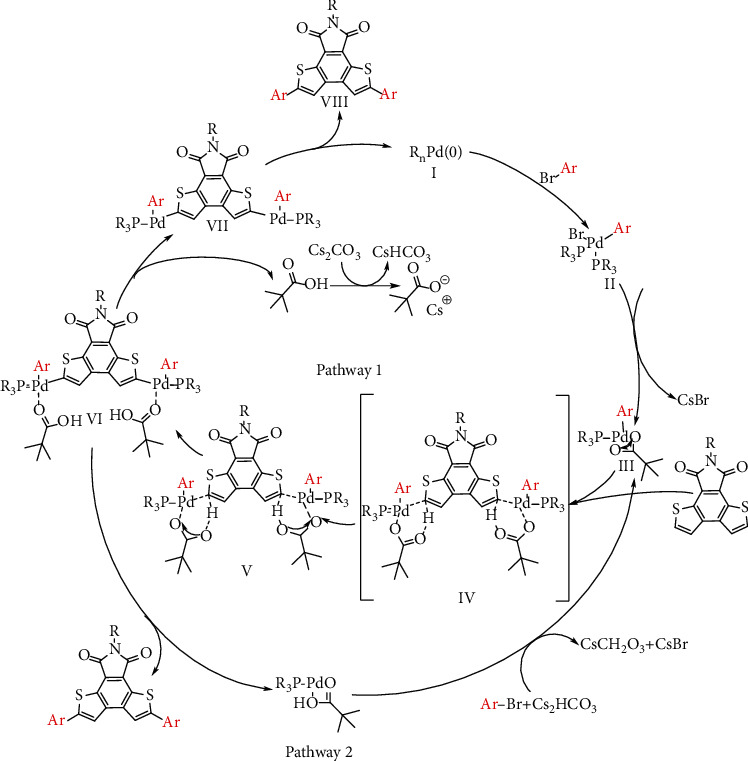
Proposed mechanism for the formation of DTI-based molecules via direct C-H arylation [63–65].

The possible mechanism for the formation of DTI-based molecules is shown in Scheme 4. The reaction mechanism studies support high regioselectivity of C-H arylation based on DTI. C-H arylation afforded *α*-diarylated DTI in high yields, while traditional Suzuki and Stille cross couplings generated mixtures of *α*- and *β*-arylated isomers due to the poor selective bromination process by NBS. The results are consistent with the reactivity profiles of electrophilic aromatic substitution via NBS bromination (Scheme S2) [60–62] but have a good correlation with a proton-transfer pathway [63–65].

**Figure 2:**
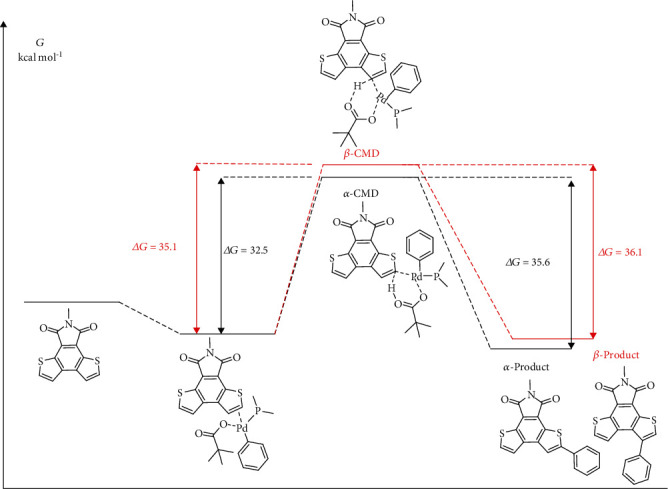
Computational study on the plausible palladium catalytic reaction of the concerted metalation-deprotonation (CMD) pathway in the gas phase.

To understand the reaction mechanism, a plausible palladium-catalyzed C-H arylation was performed (Scheme 4). The proposed mechanism begins with the brominated aromatic hydrocarbon (BrAr) onto a Pd(0) catalyst (II). Ligand exchanges generate a bidentate carboxylate complex (III). Subsequently, the DTI substrate was deprotonated by the carboxylate base, and Pd-C bond was formed, resulting in concerted metalation-deprotonation (CMD) state (IV). The carboxylic acids may lose coordination from the metal centers and react with a carbonate base (VII) through an acid-base reaction. Finally, new carbon-carbon bonds are formed via the reductive elimination (VIII), which regenerates the Pd (0) catalyst. The calculation showed the CMD state for the C-H bonds on the incipient DTI (Figure 2). The activation free energy of the *α*-position (Δ*G* = 32.5 kcal mol^−1^) of DTI is lower than that of the *β*-position (Δ*G* = 35.1 kcal mol^−1^), which indicates that the C-H bond at the *α*-position is more active than that at the *β*-position. The activation energy is lower for reactivity at the *α*-position relative to the *β*-position, which may be due to the higher reaction activity and inherent electron bias of DTI. As such, the CMD state is regioselectivity-determining. The computational studies support the regioselectivity of C-H arylation based on DTI. Moreover, the Mulliken charge distribution of DTI was calculated with DFT at the B3LYP/6-31G level (Figure S4). The result shows that the electronegativity of the carbon at *α*-position is close to that of the carbon at the *β*-position. Therefore, the electrophilic substitution of DTI with NBS bromination exhibits poor selectivity.

### 2.3. Physical Properties of DTI-Based Materials

UV-Vis absorption spectra of the DTI-based molecules were performed to explore the influence of end groups on light-harvesting performance. The analysis results of the materials are summarized in Table 2 and Figure S5. In solutions, all the compounds exhibited two obvious absorption bands at the range of 237-345 nm and 377-485 nm. The absorption bands with shorter wavelength could be attributed to localized *π*-*π*∗ transitions of the electron-rich or electron-withdrawing groups. The longer wavelength absorption bands come from the intramolecular charge transfer (ICT) transition between electron donor and electron acceptor [66, 67]. For all the DTI-based molecules, DTI2TPA showed the highest ICT energy band, while DTI without any substituted end group exhibited the lowest ICT energy band. In addition, the absorption spectra in thin film states showed significant red shift compared with those in solution states. The optical absorption edge (*λ*_onset_) of DTI, DTI2Mes, DTI2NTI, DTI2F, DTI2Py, DTI2CzR, and DTI2TPA shifted towards the longer wavelength and the optical band gap (*E*_*g*_^opt^) gradually decreased from 2.78 eV to 2.17 eV. According to the results, DTI-based molecules with stronger electron-donating end groups demonstrate a larger extent of ICT characteristics, which confirm the electron-deficient nature of the DTI unit.

**Figure 3:**
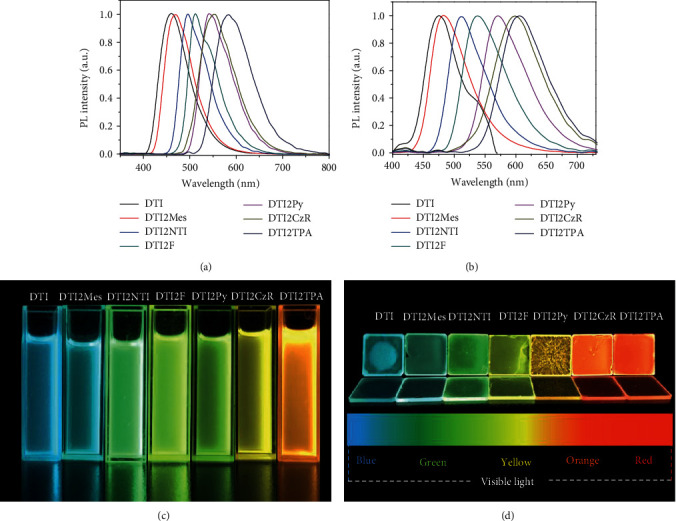
PL spectra of DTI-based molecules (a) in dilute DCM solutions (*c* = 10^−6^ mol/L) and (b) in solid films; the pictures of DTI-based materials (c) in dilute DCM solutions (*c* = 10^−6^ mol/L) and (d) in solid films under a UV lamp (365 nm).

The emissive properties of all the DTI-based molecules were measured in solutions and in films (Figures 3(a) and 3(b)). The emission maxima of DTI, DTI2Mes, DTI2NTI, DTI2F, DTI2Py, DTI2CzR, and DTI2TPA in solutions were recorded at 460, 471, 497, 512, 541, 553, and 585 nm, respectively, whereas those in films were at 475, 483, 512, 539, 570, 597, and 608 nm, respectively. A little red shift in emission spectra was observed for all the DTI-based molecules (10-20 nm) upon moving from dilute solutions to thin films. The results demonstrate that the DTI-based molecules could widely modulate the photoluminescence spectra range through cross couplings with various end groups by direct C-H arylation. As shown in Figures 3(c) and 3(d), various end group modifications on the DTI core enable the synthesis of a fluorophore library whose emission spectra cover most of the visible spectra. As shown in Table 2, much higher photoluminescence quantum yields (PLQYs) were recorded for DTI2CzR, DTI2F, and DTI2TPA as compared with those of DTI, DTI2Mes, DTI2NTI, and DTI2Py. The results show that the electron-deficient unit DTI is beneficial to the construction of a donor-acceptor-donor (D-A-D) structure and to the enhancement of ICT and luminescence. In order to further understand the photophysical properties of the obtained molecules, fluorescence decay was recorded as shown in Figure S6. The fluorescence decay times of DTI2CzR, DTI2F, and DTI2TPA in films were 4.54 ns, 2.52 ns, and 3.26 ns, which were longer than those of DTI2Mes, DTI2NTI, and DTI2Py, respectively. The results suggest that D-A-D architectures can suppress the quenching of the excited singlet states, leading to the increase of delayed fluorescence and PLQYs.

To reveal the electronic properties, electrochemical properties were studied by cyclic voltammetry (CV) (Figure S7). The results are listed in Table S2, in comparison with the calculation results with B3LYP/6-31G(d). The voltammograms revealed that the reductions were quasi reversible in most cases, but the oxidations were quite clearly irreversible in most of the voltammograms. DTI demonstrated onset oxidation potential at 1.47 V and the onset reduction potential at -1.28 V, which orresponded to the highest occupied molecular orbital (HOMO) value of -6.10 eV and the lowest occupied molecular orbital (LUMO) value of -3.33 eV, respectively. The results showed that the LUMO value of DTI was significantly lower than that of its analogue dithieno[3,2-f:2′,3′-h] phthalimide (LUMO = −3.12 eV) [68], indicating that the DTI structure was easily reduced and possessed strong electron-accepting characteristics. The results were in accordance with its electron-withdrawing capacity due to the introduction of an electron-deficient aromatic imide system. DTI-based *π*-conjugated molecules exhibited negative shifts in oxidation potentials and little shift in the reduction potentials as compared to those of the DTI unit. According to the results, the end groups of DTI showed little effect on LUMO energy levels but obviously raised the HOMO energy levels, leading to a considerable band gap reduction and a substantial red shift in the onset of absorption, which was consistent with the trend based on optical band gaps (*E*_*g*_^opt^) and theoretical band gaps (*E*_*g*_^cal^).

**Figure 4:**
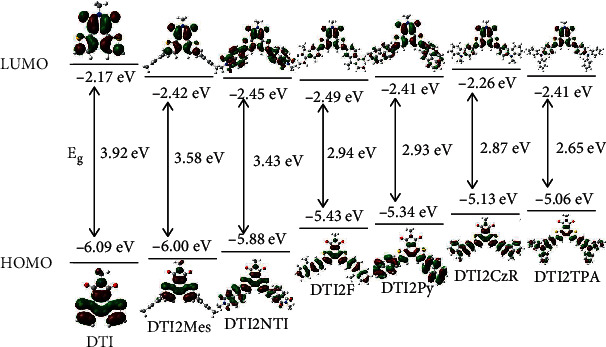
The calculated electron cloud distribution of DTI-based molecules with DFT at the B3LYP/6-31G level.

To better understand the effect of end groups, DFT calculations were performed on all the molecules. The optimized geometries of all the investigated molecules show planar DTI cores, which is preferred for intermolecular *π*-*π* stacking. The torsion angle between the end groups and the core for DTI2Mes is 90° owing to the extra bulkiness of mesitylene (Figure S8). However, the geometry of DTI2F and DTI2CzR is almost planar, which increases the conjugation of these molecules. Based on the predicted ground state structure with global minimum energies, the time-dependent (TD) DFT simulation was carried out by TD-B3LYP/6-31G, and the optical and electronic properties of DTI and its derivatives were calculated (Figure 4). Most of the molecules show extensively delocalized HOMO and LUMO spatial distributions, except for DTI2Mes that shows vertical structure between DTI and mesitylene unit. There is little overlap between HOMO and LUMO for DTI2CzR and DTI2TPA, indicating that large intramolecular charger transfer occurs. Despite the comparable torsion angles, the aryl substituents had a great impact on the energy levels of DTI, gradually raising the HOMO energy level from -6.09 eV to -5.06 eV. The trend of the theoretical band gap and HOMO energy levels for DTI-based molecules was similar to that of CV measurements.

### 2.4. OLED Performance of DTI2F and DTI2CzR

**Figure 5:**
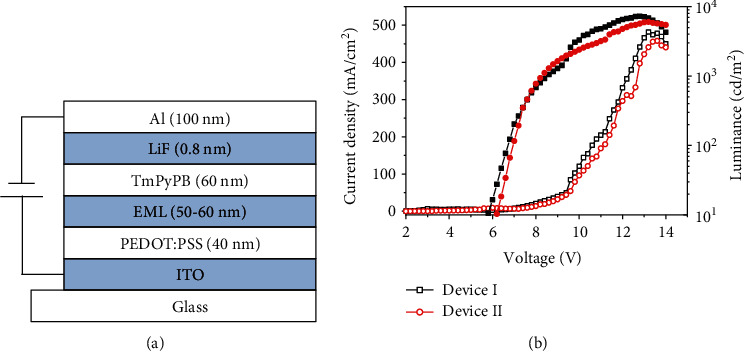
(a) OLED configuration and (b) current density-voltage (open symbols) and luminance-voltage (solid symbols) curve characteristics of the devices based on DTI2F and DTI2CzR.

With achieving excellent solid-state fluorescence and high PLQYs of DTI2F and DTI2CzR, the potential application of these molecules in OLEDs was investigated. A set of unoptimized OLEDs based on DTI2F (device I) and DTI2CzR (device II) was fabricated by solution processing. A single-emissive-layer OLED with the configuration ITO/PEDOT:PSS (40 nm)/DTI2F or DTI2CzR/TmPyPB (60 nm)/LiF (0.8 nm)/Al (100 nm) was fabricated (Figure 5(a)). PEDOT:PSS was spin-coated on ITO substrates and annealed at 150°C for 60 min. DTI2F and DTI2CzR film was coated onto PEDOT:PSS film with a thickness of 50-60 nm from toluene solution. Then, TmPyPB, LiF, and Al were consecutively deposited onto the emission layer. The EL spectra of devices I and II are shown in Figure S9, and the maximum emission peaks at 535 nm and 590 nm for DTI2F and DTI2CzR, respectively, which are similar to their photoluminescence data in solid films. As shown in Figure 5(b), the maximum brightness of device I is 7200 cd/m^2^ at 11 V, which is higher than that of device II (6100 cd/m^2^ at 11 V). The maximum current efficient is 3.8 cd/A and 3.2 cd/A for devices I and II, respectively (Figure S10). The simple device structures demonstrate good OLED performances, indicating that direct C-H arylation is feasible and powerful to construct DTI-based molecules as promising active materials for organic optoelectronic applications.

## 3. Discussion

In this work, we have developed a facile selective direct C-H arylation methodology for efficient construction of a variety of symmetrical DTI-based *π*-conjugated molecules. The resulting methodology is effective and applicable for a wide range of substrates from electron-rich units to electron-deficient units with large steric end groups. In addition, direct C-H arylation of DTI is highly selective and ecofriendly as compared with the traditional Suzuki or Stille cross-coupling routes. Aryl halides have been confirmed to be able to couple with DTI via direct C-H arylation, showing high regioselectivity. According to this green methodology, various symmetrical new conjugated molecules based on DTI were successfully synthesized in a one-pot procedure, affording good to excellent yields. Varying the functional end groups onto the DTI core has been demonstrated to fine tune the emission colors to cover most of the visible spectra, which are potential active materials for organic optoelectronics, i.e., OLEDs. According to these photophysical and eletrochemical properties, the conjugated molecules are good candidates for high-performance solution-processable optoelectronic devices. Preliminary OLED measurements show that devices based on DTI2F and DTI2CzR achieved a maximum brightness of 7200 cd/m^2^ and 6100 cd/m^2^, respectively. The results suggest a facile strategy towards highly selective direct C-H arylation for the efficient construction of *π*-conjugated molecules for organic optoelectronic applications.

## Figures and Tables

**Table 1:** Conditions and yields for the direct C-H arylation with various brominated substituents.

Entry	Compound	Br-Ar	[Pd]^a^	Ligand^a^	Base^a^	Solvent^a^	Yield (%)
1	DTI2Mes	BrMes	Pd(OAc)_2_	PCy_3_	K_2_CO_3_	THF	Trace
2	DTI2Mes	BrMes	Pd(OAc)_2_	PPh_3_	K_2_CO_3_	THF	14
3	DTI2Mes	BrMes	Pd(OAc)_2_	P(*o*-Tol)_3_	K_2_CO_3_	THF	19
4	DTI2Mes	BrMes	Pd(OAc)_2_	P(2-MeOPh)_3_	K_2_CO_3_	THF	30
5	DTI2Mes	BrMes	Pd(OAc)_2_	P(2-MeOPh)_3_	Cs_2_CO_3_	THF	33
6	DTI2Mes	BrMes	Pd_2_(dba)_3_	P(2-MeOPh)_3_	Cs_2_CO_3_	THF	44
7	DTI2Mes	BrMes	Pd_2_(dba)_3_	P(2-MeOPh)_3_	Cs_2_CO_3_	DMF	50
8	DTI2Mes	BrMes	Pd_2_(dba)_3_	P(2-MeOPh)_3_	Cs_2_CO_3_	Toluene	63
9	DTI2Mes	BrMes	Pd_2_(dba)_3_	P(2-MeOPh)_3_	Cs_2_CO_3_	*o*-Xylene	84
10	DTI2Cz	BrCz	Pd_2_(dba)_3_	P(2-MeOPh)_3_	Cs_2_CO_3_	*o*-Xylene	Trace
11	DTI2CzR	BrCzR	Pd_2_(dba)_3_	P(2-MeOPh)_3_	Cs_2_CO_3_	*o*-Xylene	58
12	DTI2TPA	BrTPA	Pd_2_(dba)_3_	P(2-MeOPh)_3_	Cs_2_CO_3_	*o*-Xylene	72
13	DTI2F	BrF	Pd_2_(dba)_3_	P(2-MeOPh)_3_	Cs_2_CO_3_	*o*-Xylene	61
14	DTI2Th	BrTh	Pd_2_(dba)_3_	P(2-MeOPh)_3_	Cs_2_CO_3_	*o*-Xylene	Trace
15	DTI2Py	BrPy	Pd_2_(dba)_3_	P(2-MeOPh)_3_	Cs_2_CO_3_	*o*-Xylene	54
16	DTI2PDI	BrPDI	Pd_2_(dba)_3_	P(2-MeOPh)_3_	Cs_2_CO_3_	*o*-Xylene	Trace
17	DTI2DPP	BrDPP	Pd_2_(dba)_3_	P(2-MeOPh)_3_	Cs_2_CO_3_	*o*-Xylene	Trace
18	DTI2NTI	BrNTI	Pd_2_(dba)_3_	P(2-MeOPh)_3_	Cs_2_CO_3_	*o*-Xylene	63

^a^The structures of ligands and palladium catalysts are shown in Figure S1. DTI (41.3 mg, 0.1 mmol, 1 equiv.), [Pd] (3 mol%), ligand (12 mol%), base (3.0 equiv.), pivalic acid (1 equiv.), and 0.1 M of THF in 85°C, DMF in 110°C, toluene in 100°C. and *o*-xylene in 120°C. Isolated yield was obtained from column chromatography.

**Table 2:** Photophysical properties of DTI-based materials.

	*λ*_abs,solution_ (nm)	*λ*_abs,film_ (nm)	*λ*_onset_ (nm)	*E*_*g*_^opt^ (eV)	*λ*_PL,solution_ (nm)	*λ*_PL,film_ (nm)	*τ*_solution_ (ns)	*τ*_film_ (ns)	PLQY (%)
DTI	271, 377	274, 386	445	2.78	460	475	3.25	2.66	10
DTI2Mes	262, 382	265, 392	450	2.75	471	483	1.57	2.19	17
DTI2NTI	237, 423	259, 437	489	2.53	497	512	1.29	1.16	31
DTI2F	334, 449	336, 455	521	2.38	512	539	1.44	2.52	59
DTI2Py	345, 444	354, 460	537	2.31	541	570	1.37	1.43	16
DTI2CzR	303, 468	310, 487	552	2.24	553	597	4.84	4.54	67
DTI2TPA	302, 485	306, 496	570	2.17	585	608	4.33	3.26	31

## Supplementary Materials

Supplementary Materials Figure S1: structures of commonly used palladium catalysts and ligands in direct C-H arylation. Scheme S1: synthesis of DTI. Scheme S2: brominated reaction mechanism of DTI in the presence of NBS. Figure S2: the experimental and theoretical ^1^H NMR spectra of 2BrDTI. Table S1: crystallographic data for DTI2Mes. Figure S3: oak ridge thermal ellipsoid plot (ORTEP) view of the molecular structures and packing structure of DTI2Mes. Figure S4: the Mulliken charge distribution of DTI. Figure S5: (a) UV-Vis absorption of DTI-based conjugated molecules in dilute DCM solutions (10-6 M) and (b) in the films spin-cast on quartz. Figure S6: (a) fluorescent lifetime tests of DTI-based conjugated molecules in dilute DCM olutions (10-6 M) and (b) in the spin-coated films. Table S2: CV data for DTI-based conjugated molecules. Figure S7: cyclic voltammogram (CV) curves of DTI-based molecules. Figure S8: side views of the optimized geometries obtained from DFT calculations at the B3LYP/6-31G level. Figure S9: electroluminescence (EL) spectra of the devices based on DTI2F and DTI2CzR. Figure S10: (a) current efficiency-current density curves and (b) EQE-current density curves of the devices based on DTI2F and DTI2CzR. Figures S11-S32: MALDI-TOF mass spectra, ^1^H NMR, and 13C NMR of the synthesized compounds.
