# Supplementary material for: Highly Regioselective Direct C-H Arylation: Facile Construction of Symmetrical Dithienophthalimide-Based π-Conjugated Molecules for Optoelectronics
Source: Research (Wash D C). 2020 Aug 30;2020:9075697. doi: 10.34133/2020/9075697 (PMC7510346; doi:10.34133/2020/9075697)
Supplement: Supplementary Materials — Figure S1: structures of commonly used palladium catalysts and ligands in direct C-H arylation. Scheme S1: synthesis of DTI. Scheme S2: brominated reaction mechanism of DTI in the presence of NBS. Figure S2: the experimental and theoretical 1H NMR spectra of 2BrDTI. Table S1: crystallographic data for DTI2Mes. Figure S3: oak ridge thermal ellipsoid plot (ORTEP) view of the molecular structures and packing structure of DTI2Mes. Figure S4: the Mulliken charge distribution of DTI. Figure S5: (a) UV-Vis absorption of DTI-based conjugated molecules in dilute DCM solutions (10-6 M) and (b) in the films spin-cast on quartz. Figure S6: (a) fluorescent lifetime tests of DTI-based conjugated molecules in dilute DCM olutions (10-6 M) and (b) in the spin-coated films. Table S2: CV data for DTI-based conjugated molecules. Figure S7: cyclic voltammogram (CV) curves of DTI-based molecules. Figure S8: side views of the optimized geometries obtained from DFT calculations at the B3LYP/6-31G level. Figure S9: electroluminescence (EL) spectra of the devices based on DTI2F and DTI2CzR. Figure S10: (a) current efficiency-current density curves and (b) EQE-current density curves of the devices based on DTI2F and DTI2CzR. Figures S11-S32: MALDI-TOF mass spectra, 1H NMR, and 13C NMR of the synthesized compounds. [file 9075697.f1.pdf]

Supplementary Materials

Highly Regioselective Direct C-H Arylation: Facile Construction of Symmetrical Dithienophthalimide-Based π-Conjugated Molecules for Optoelectronics

Xiang-Chun Li,[‡,a] Yibo Xue,[‡,a] Wan Song,[a] Yu Yan,[a] Jie Min,[a] Fang Liu,[a] Xu Liu,[a]

Wen-Yong Lai,*[a,b] and Wei Huang[a,b]

[a] Key Laboratory for Organic Electronics and Information Displays & Institute of Advanced Materials (IAM), Nanjing University of Posts & Telecommunications, 9 Wenyuan Road, Nanjing 210023, China

[b] Frontiers Science Center for Flexible Electronics, Xi'an Institute of Flexible Electronics (IFE) and Xi'an Institute of Biomedical Materials & Engineering, Northwestern Polytechnical University, 127 West Youyi Road, Xi'an 710072, China

*E-mail: iamwylai@njupt.edu.cn

[‡]These authors contributed equally to this work.

1. Materials and methods

General Methods. The matrix assisted laser desorption ionization time of flight mass spectroscopy (MALDI-TOF MS) measurements were carried out with a Bruker AV400 mass spectrometer. NMR spectra were recorded on a Bruker Ultra Shield Plus 400 MHz NMR (^{1}H: 400 MHz, ^{13}C: 100 MHz). The ^{1}H NMR spectra of **2BrDTI** were calculated using GGA: PBE method with TZP basis set by ADFjobs. UV-Visible absorption spectra were recorded on a PerkinElmer Lambda 35. Photoluminescence (PL) spectra were measured using a PerkinElmer LS55. The fluorescence quantum yields were determined by full-featured steady state/transient fluorescence spectrometer FLS-920 from Edinburgh Instruments. PL decays were measured with an Edinburgh FLS-920 spectrometer. All fluorescent lifetimes were determined from the data using the Edinburgh Instruments software package. Electrochemical behaviors were investigated by cyclic voltammetry (CV) with a standard three-electrode electrochemical cell in a 0.1 M tetra-*n*-butylammonium hexafluorophosphate (Bu_4NPF_6) in nonaqueous acetonitrile at room temperature under nitrogen with a scanning rate of 50 mV/s. A platinum working electrode, a glassy carbon electrode, and an Ag/$AgNO_3$ (0.1 M) reference electrode were used. The single-crystal XRD were measured using a Bruker D8 Quest. The CV curves were calibrated using ferrocene/ferrocenium (Fc/Fc^{+}) redox couple (4.80 eV below the vacuum level) as the internal standard. Thus, the highest occupied molecular orbital (HOMO) and the lowest unoccupied molecular orbital (LUMO) energy levels could be calculated according to: $E_{\mathrm{HOMO}} = -(E_{\mathrm{ox}}-E_{\mathrm{Fc}}+4.8)$ eV and $E_{\mathrm{LUMO}} = -(E_{\mathrm{red}}-E_{\mathrm{Fc}}+4.8)$ eV, where E_{ox} and E_{red} were determined from the onset of the first potential of the oxidation and reduction curves, respectively, and E_{Fc} was taken as the half-wave potential of ferrocene. The molecular structures were optimized using density functional theory (DFT) method (B3LYP) with 6-31G (d) basis set by GAUSSIAN 09.

Device Fabrication and Testing. The patterned indium tin oxide (ITO) glass substrates were ultrasonically cleaned with detergent, alcohol and acetone, deionized water and then dried at 120 ℃ in a vacuum oven for more than 1 h. After the ultraviolet (UV)-ozone treatment for 4 min, a 20 nm PEDOT:PSS was spin-coated onto the ITO substrates and dried at 120 ℃ in a vacuum oven for 15 min to remove the residue solvent. The samples were then moved into a glove box under a nitrogen-protected environment, and the emissive layer (EML) was spin-coated on top of PEDOT:PSS from chloroform and then annealed using a hot plate at 80 ℃ for 20 min to extract the residual solvent. Following that, the samples were transferred to a thermal evaporator chamber connected to the glove box without exposure to the atmosphere. 60 nm TmPyPB, 0.8 nm LiF, and 100 nm Al were sequentially deposited by thermal evaporation under a pressure of 5×10^{-4} Pa. The luminance-current-voltage characteristics of the devices were recorded using a combination of a Keithley source-meter (model 2602) and a calibrated luminance meter. Electroluminescence (EL) spectra were obtained using a spectra-scan PR655 spectrophotometer. The thickness of the organic films was measured by using a spectroscopic ellipsometry (α-SE, J.A. Wollam Co. Inc.). All the measurements were carried out at room temperature under ambient conditions.

Materials. All the reagents used were purchased from Sigma-Aldrich, J&K or Xiya Reagent (China). When necessary, solvents and reagents were purified using standard procedures.

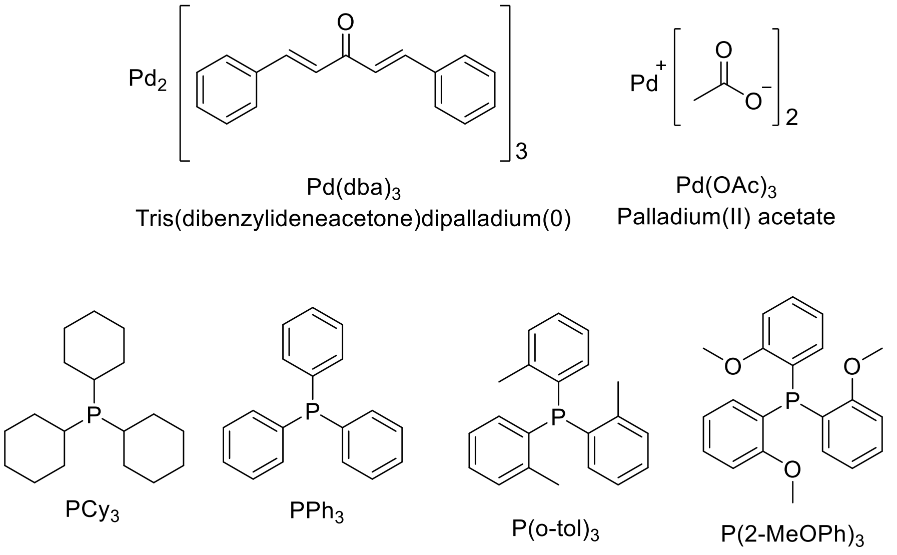

Figure S1. Structures of commonly used palladium catalysts and ligands in direct C-H arylation.

2. Synthetic procedures

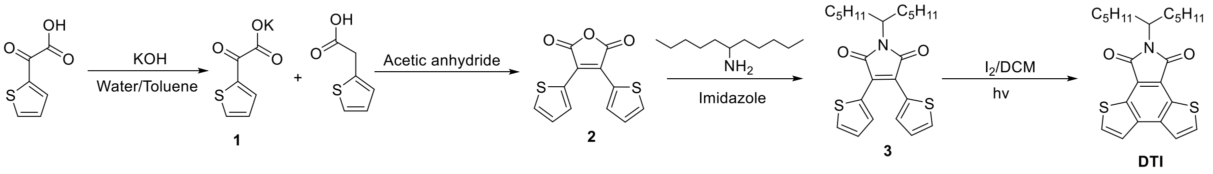

Scheme S1. Synthesis of **DTI**.

Potassium 2-oxo-2-(thiophen-2-yl)acetate (1): To a mixture of 2-oxo-2-(thiophen-2-yl) acetic acid (1.0 g, 6.4 mmol) and KOH (359 mg, 6.4 mmol) in toluene (10 mL), water (5 mL) was added and stirred at 100 ℃ for 1 h. After cooling to room temperature, the reaction mixture was concentrated in vacuo and dried. The product was obtained with 98% yield. ^{1}H NMR (400 MHz, DMSO-d6, *δ*, ppm): 7.89 (dd, *J* = 4.9, 0.8 Hz, 1H), 7.71 (dd, *J* = 3.7, 0.8 Hz, 1H), 7.17 (dd, *J* = 4.7, 3.9 Hz, 1H).

3,4-di(thiophen-2-yl)furan-2,5-dione (2): To a mixture of potassium 2-oxo-2-(thiophen-2-yl) acetate (**1**) (1.15 g, 5.9 mmol) and 2-(thiophen-2-yl)acetic acid (925 mg, 6.5 mmol), dry acetic anhydride (20 mL) was added and stirred at 100 ℃ for 1 h. After cooling, the reaction mixture was concentrated in vacumn. The residue was dissolved in ethyl acetate (50 mL) and washed twice with water. The organic phase was dried with $MgSO_4$. The crude product was purified by column

chromatography on silica gel (DCM/Hexane, 3:1 v/v) to afford **2** (1.5 g, 86%). ^{1}H NMR (400 MHz, $CDCl_3$, δ, ppm): 7.97 (dd, J = 3.8, 1.0 Hz, 2H), 7.67 (dd, J = 5.1, 1.0 Hz, 2H), 7.19 (dd, J = 5.0, 3.9 Hz, 2H).

3,4-di(thiophen-2-yl)-1-(undecan-6-yl)-1H-pyrrole-2,5-dione (3): To a 50 mL round bottom flask was added 3,4-di(thiophen-2-yl)furan-2,5-dione (**2**) (1.0 g, 3.93 mmol) and imidazole (4.0 g, 5.8 mmol). Then, 3.0 mL of undecan-6-amine was added. The reaction mixture was refluxed at 120 ℃ for 2 h under N_2. After cooling to room temperature, the product was extracted into ethyl acetate, and the organic layers were then collected and dried with $MgSO_4$. The product was recrystallized by DCM and methanol and reacted next without further purification.

8-(Undecan-6-yl)-7H-dithieno[2,3-e:3',2'-g]isoindole-7,9(8H)-dione (DTI): A solution of 3,4-di(thiophen-2-yl)-1-(undecan-6-yl)-1H-pyrrole-2,5-dione (**3**) (1.0 g, 2.41 mmol) dissolved in CH_2Cl_2 (90 mL) in the presence of a catalytic amount of I_2 (478 mg, 3.8 mmol) was irradiated for 10 h in a reactor with an HPK-125W Philips high-pressure mercury vapor lamp in a water-jacketed immersion well. The solvent was evaporated and the residue was purified by chromatography on silica gel (DCM/Hexane, 1:4 v/v) to afford **DTI** (875.9 mg, 88%). ^{1}H NMR (400 MHz, $CDCl_3$, δ, ppm): 7.94 (d, J = 5.4 Hz, 2H), 7.85 (d, J = 5.4 Hz, 2H), 4.30-4.22 (m, 1H), 2.15 (ddd, J = 19.1, 12.1, 7.2 Hz, 2H), 1.81-1.72 (m, 2H), 1.35-1.25 (m, 12H), 0.85 (t, J = 7.0 Hz, 6H). ^{13}C NMR (101 MHz, $CDCl_3$, δ, ppm): 169.04, 139.95, 132.74, 129.86, 123.15, 121.71, 52.66, 32.73, 31.54, 26.49, 22.53, 14.03. Anal. Calcd for $C_{23}H_{27}NO_2S_2$: C, 66.79; H, 6.58; N, 3.39. Found: C, 66.52; H, 6.31; N, 3.64.

General Procedure for the Direct C-H Arylation of DTI

To a solution of $Pd_2(dba)_3$ (3 mol%), ligand (12 mol%), base (3.0 mmol) and PivOH (1.0 mmol) in *o*-xlyene (20 mL) in a dried Schlenk tube were added **DTI** (1.0 mmol) and the aryl bromides (3.0

mmol) under N_2. The reaction mixture was then heated at 120°C under N_2 for 24 h. After the reaction mixture had cooled to room temperature, water was added. The product was extracted into DCM, and the organic layers were subsequently concentrated in vacumn and dried with $MgSO_4$. Purification by flash chromatography (DCM/Hexane) yielded the desired product.

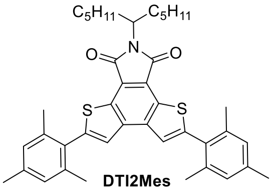

2,5-Dimesityl-8-(undecan-6-yl)-7H-dithieno[2,3-e:3',2'-g]isoindole-7,9(8H)-dione (DTI2Mes): Under nitrogen, **DTI** (82.7 mg, 0.2 mmol), bromomesitylene (**BrMes**, 118.8 mg, 0.6 mmol), Cs_2CO_3 (195.5 mg, 0.6 mmol), P(2-MeOPh)$_3$ (8.4 mg, 0.024 mmol), PivOH (20.4 mg, 0.2 mmol)), and $Pd_2(dba)_3$ (5.5 mg, 0.006 mmol) were dissolved in *o*-xlyene (12 mL) and the solution was refluxed at 120°C for 24 h. The product was extracted into DCM, and the resulting organic layers were collected and dried with $MgSO_4$. The crude product was purified by column chromatography on silica gel (DCM/Hexane, 1:5 v/v) to afford **DTI2Mes** (109.1 mg, 84%). ^{1}H NMR (400 MHz, $CDCl_3$, δ, ppm): 8.75-8.58 (m, 14H), 7.48 (s, 2H), 7.03 (s, 4H), 4.30-4.23 (m, 1H), 2.39 (s, 6H), 2.23 (s, 12H), 2.16 (dd, J = 9.1, 4.7 Hz, 2H), 1.81-1.72 (m, 2H), 1.36-1.27 (m, 12H), 0.87 (t, J = 7.0 Hz, 6H). ^{13}C NMR (101 MHz, $CDCl_3$, δ, ppm): 169.18, 149.64, 140.19, 138.88, 137.72, 130.87, 130.01, 128.38, 122.38, 121.08, 52.68, 32.78, 31.59, 29.72, 26.54, 22.56, 21.16, 20.68, 14.05. Calcd for MS: 649.30, Found: (M^+). 649.46. Anal. Calcd for $C_{41}H_{47}NO_2S_2$: C, 75.77; H, 7.29; N, 2.16. Found: C, 75.51; H, 7.65; N, 2.42.

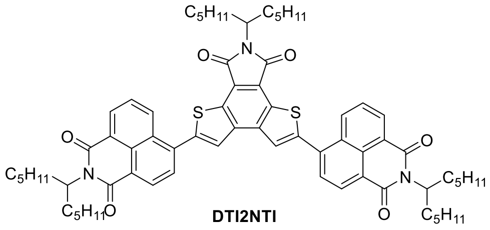

6,6'-(7,9-Dioxo-8-(undecan-6-yl)-8,9-dihydro-7H-dithieno[2,3-e:3',2'-g]isoindole-2,5-diyl)bis

(2-(undecan-6-yl)-1H-benzo[de]isoquinoline-1,3(2H)-dione) (DTI2NTI): Under nitrogen, **DTI** (82.7 mg, 0.2 mmol), 6-bromo-2-(undecan-6-yl)-1H-benzo[de]isoquinoline-1,3(2H)-dione (**BrNTI**, 257.5 mg, 0.6 mmol), Cs_2CO_3 (195.5 mg, 0.6 mmol), P(2-MeOPh)$_3$ (8.4 mg, 0.024 mmol), PivOH (20.4 mg, 0.2 mmol)), and Pd_2(dba)$_3$ (5.5 mg, 0.006 mmol) were dissolved in *o*-xlyene (12 mL) and the solution was refluxed at 120°C for 24 h. The product was extracted into DCM, and the resulting organic layers were collected and dried with $MgSO_4$. The crude product was purified by column chromatography on silica gel (DCM/Hexane, 1:4 v/v) to afford **DTI2NTI** (140.0 mg, 63%). ^{1}H NMR(400 MHz, $CDCl_3$, δ, ppm): 8.69 (d, J = 8.4 Hz, 6H), 8.10 (s, 2H), 8.04 (d, J = 7.6 Hz, 2H), 7.88-7.81 (m, 2H), 5.26-5.18 (m, 2H), 4.35-4.28 (m, 1H), 2.26 (dd, J = 15.4, 6.0 Hz, 6H), 1.93-1.79 (m, 6H), 1.31 (dd, J = 16.1, 8.3 Hz, 36H), 0.88 (dd, J = 12.8, 6.6 Hz, 18H). ^{13}C NMR (101 MHz, $CDCl_3$, δ, ppm): 168.63, 147.30, 144.89, 140.06, 137.33, 131.28, 129.87, 129.37, 128.95, 127.88, 123.24, 122.92, 54.74, 53.00, 32.71, 32.34, 31.75, 31.53, 29.71, 26.56, 22.56, 14.03. Calcd for MS: 1112.54, Found: (M^+). 1112.26. Anal. Calcd for $C_{69}H_{81}N_3O_6S_2$: C, 74.49; H, 7.34; N, 3.78. Found: C, 74.84; H, 7.58; N, 3.51.

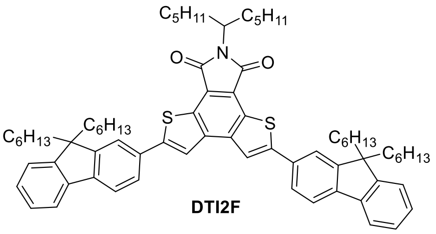

DTI2F

2,5-Bis(9,9-dihexyl-9H-fluoren-2-yl)-8-(undecan-6-yl)-7H-dithieno[2,3-e:3',2'-g]isoindole-7,9(8H)-dione (DTI2F): Under nitrogen, **DTI** (82.7 mg, 0.2 mmol), 2-bromo-9,9-dihexyl-9H-fluorene (**BrF**, 247.3 mg, 0.6 mmol), Cs_2CO_3 (195.5 mg, 0.6 mmol), P(2-MeOPh)$_3$ (8.4 mg, 0.024 mmol), PivOH (20.4 mg, 0.2 mmol)), and Pd_2(dba)$_3$ (5.5 mg, 0.006 mmol) were dissolved in *o*-xlyene (12 mL) and the solution was refluxed at 120°C for 24 h. The product was extracted into DCM, and the resulting organic layers were collected and dried with $MgSO_4$. The crude product was purified by

column chromatography on silica gel (DCM/Hexane, 1:6 v/v) to afford **DTI2F** (131.5 mg, 61%). ^{1}H NMR(400 MHz, $CDCl_3$, δ, ppm): 8.12 (s, 2H), 7.88 (d, J = 8.1 Hz, 2H), 7.83-7.79 (m, 4H), 7.76 (d, J = 5.0 Hz, 2H), 7.38 (dd, J = 6.6, 3.7 Hz, 6H), 4.28 (s, 1H), 2.09-2.03 (m, 8H), 1.18-1.03 (m, 48H), 0.78 (dd, J = 7.7, 4.6 Hz, 18H). ^{13}C NMR (101 MHz, $CDCl_3$, δ, ppm): 169.09, 151.94, 151.84, 151.17, 142.67, 140.59, 140.22, 132.15, 129.74, 127.76, 127.01, 125.67, 123.02, 122.25, 121.07, 120.37, 120.10, 116.62, 55.37, 52.62, 40.44, 32.76, 31.94, 31.57, 31.49, 31.45, 30.21, 29.71, 29.37, 26.49, 23.80, 22.70, 22.58, 19.74, 14.12, 14.04, 13.99. Calcd for MS: 1077.65, Found: (M^+). 1078.43. Anal. Calcd for $C_{73}H_{91}NO_2S_2$: C, 81.29; H, 8.50; N, 1.30. Found: C, 81.52; H, 8.34; N, 1.48.

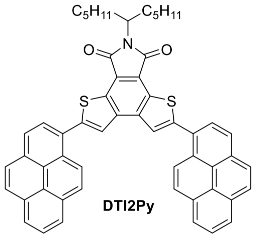

DTI2Py

2,5-Di(pyren-4-yl)-8-(undecan-6-yl)-7H-dithieno[2,3-e:3',2'-g]isoindole-7,9(8H)-dione (DTI2Py): Under nitrogen, **DTI** (82.7 mg, 0.2 mmol), 1-bromopyrene (**BrPy**, 168.0 mg, 0.6 mmol), Cs_2CO_3 (195.5 mg, 0.6 mmol), P(2-MeOPh)$_3$ (8.4 mg, 0.024 mmol), PivOH (20.4 mg, 0.2 mmol)), and $Pd_2(dba)_3$ (5.5 mg, 0.006 mmol) were dissolved in *o*-xlyene (12 mL) and the solution was refluxed at 120°C for 24 h. The product was extracted into DCM, and the resulting organic layers were collected and dried with $MgSO_4$. The crude product was purified by column chromatography on silica gel (DCM/Hexane, 1:4 v/v) to afford **DTI2Py** (87.8 mg, 54%). ^{1}H NMR(400 MHz, $CDCl_3$, δ, ppm): 8.62 (d, J = 9.3 Hz, 2H), 8.30-8.19 (m, 8H), 8.19-8.09 (m, 8H), 8.05 (t, J = 7.6 Hz, 2H), 4.31 (dd, J = 10.1, 5.2 Hz, 1H), 2.26-2.16 (m, 2H), 1.80 (d, J = 6.2 Hz, 2H), 1.35 (dd, J = 21.4, 6.6 Hz, 12H), 0.88 (t, J = 6.8 Hz, 6H). ^{13}C NMR (101 MHz, $CDCl_3$, δ, ppm): 169.12, 150.00, 140.36, 131.93, 131.43, 131.09, 130.86, 129.24, 128.75, 128.58, 128.54,

128.47, 127.33, 126.42, 125.88, 125.55, 125.05, 124.77, 124.66, 124.47, 122.55, 122.35, 52.78, 32.81, 31.61, 26.58, 22.59, 14.07. Calcd for MS: 813.27, Found: (M^+). 813.33. Anal. Calcd for $C_{55}H_{43}NO_2S_2$: C, 81.15; H, 5.32; N, 1.72. Found: C, 81.41; H, 5.15; N, 1.61.

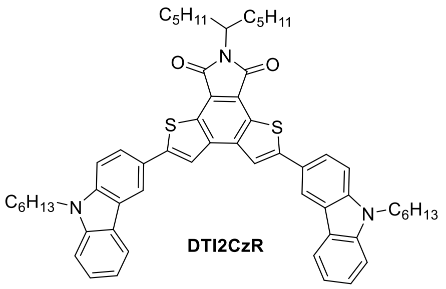

2,5-Dimesityl-8-(undecan-6-yl)-7H-dithieno[2,3-e:3',2'-g]isoindole-7,9(8H)-dione (DTI2CzR): Under nitrogen, **DTI** (82.7 mg, 0.2 mmol), 3-bromo-9-hexyl-9H-carbazole (**BrCzR**, 197.4 mg, 0.6 mmol), Cs_2CO_3 (195.5 mg, 0.6 mmol), $P(2\text{-}MeOPh)_3$ (8.4 mg, 0.024 mmol), PivOH (20.4 mg, 0.2 mmol)), and $Pd_2(dba)_3$ (5.5 mg, 0.006 mmol) were dissolved in *o*-xlyene (12 mL) and the solution was refluxed at 120°C for 24 h. The product was extracted into DCM, and the resulting organic layers were collected and dried with $MgSO_4$. The crude product was purified by column chromatography on silica gel (DCM/Hexane, 1:5 v/v) to afford **DTI2CzR** (105.7 mg, 58%). ^1H NMR(400 MHz, $CDCl_3$, δ, ppm): 8.59 (d, J = 1.4 Hz, 2H), 8.20 (d, J = 7.6 Hz, 2H), 8.06 (s, 2H), 7.98 (dd, J = 8.5, 1.7 Hz, 2H), 7.56-7.45 (m, 6H), 7.32 (t, J = 7.4 Hz, 2H), 4.38-4.29 (m, 5H), 2.26-2.17 (m, 2H), 1.97-1.89 (m, 4H), 1.81 (d, J = 5.8 Hz, 2H), 1.36 (dd, J = 10.8, 7.1 Hz, 24H), 0.92-0.87 (m, 12H). ^{13}C NMR (101 MHz, $CDCl_3$, δ, ppm): 169.22, 152.27, 141.05, 140.97, 140.65, 129.52, 126.36, 124.59, 124.56, 123.53, 122.74, 121.77, 120.60, 119.53, 118.82, 115.52, 109.30, 109.12, 52.52, 43.33, 32.81, 31.62, 31.58, 29.71, 29.00, 26.99, 26.54, 22.58, 14.07, 14.02. Calcd for MS: 912.31, Found: (M^+). 912.45. Anal. Calcd for $C_{59}H_{65}N_3O_2S_2$: C, 77.68; H, 7.18; N, 4.61. Found: C, 77.81; H, 6.92; N, 4.42.

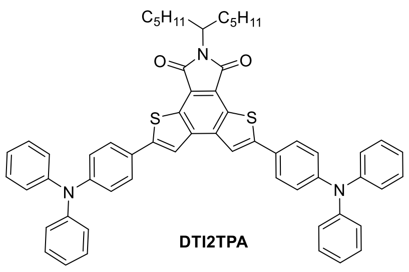

2,5-Bis(4-(diphenylamino)phenyl)-8-(undecan-6-yl)-7H-dithieno[2,3-e:3',2'-g]isoindole-7,9(8H)-dione (DTI2TPA): Under nitrogen, **DTI** (82.7 mg, 0.2 mmol), 4-bromo-*N,N*-diphenylaniline (**BrTPA**, 193.8 mg, 0.6 mmol), Cs_2CO_3 (195.5 mg, 0.6 mmol), P(2-MeOPh)$_3$ (8.4 mg, 0.024 mmol), PivOH (20.4 mg, 0.2 mmol)), and Pd_2(dba)$_3$ (5.5 mg, 0.006 mmol) were dissolved in *o*-xlyene (12 mL) and the solution was refluxed at 120°C for 24 h. The product was extracted into DCM, and the resulting organic layers were collected and dried with $MgSO_4$. The crude product was purified by column chromatography on silica gel (DCM/Hexane, 1:4 v/v) to afford **DTI2TPA** (129.5 mg, 72%). ^{1}H NMR (400 MHz, $CDCl_3$, δ, ppm): 7.90 (s, 2H), 7.70 (d, J = 8.8 Hz, 4H), 7.34 (dd, J = 8.3, 7.4 Hz, 8H), 7.16 (ddd, J = 21.7, 10.8, 4.2 Hz, 16H), 4.29-4.21 (m, 1H), 2.21-2.11 (m, 2H), 1.76 (dd, J = 13.8, 4.7 Hz, 2H), 1.35-1.27 (m, 12H), 0.86 (t, J = 6.9 Hz, 6H). ^{13}C NMR (101 MHz, $CDCl_3$, δ, ppm): 169.07, 150.80, 148.99, 147.09, 140.49, 129.51, 127.51, 126.72, 125.18, 123.83, 122.61, 121.94, 115.66, 52.56, 32.75, 31.57, 26.50, 22.55, 14.05. Calcd for MS: 899.36, Found: (M^+). 900.32. Anal. Calcd for $C_{59}H_{53}N_3O_2S_2$: C, 78.72; H, 5.93; N, 4.67. Found: C, 79.01; H, 5.80; N, 4.73.

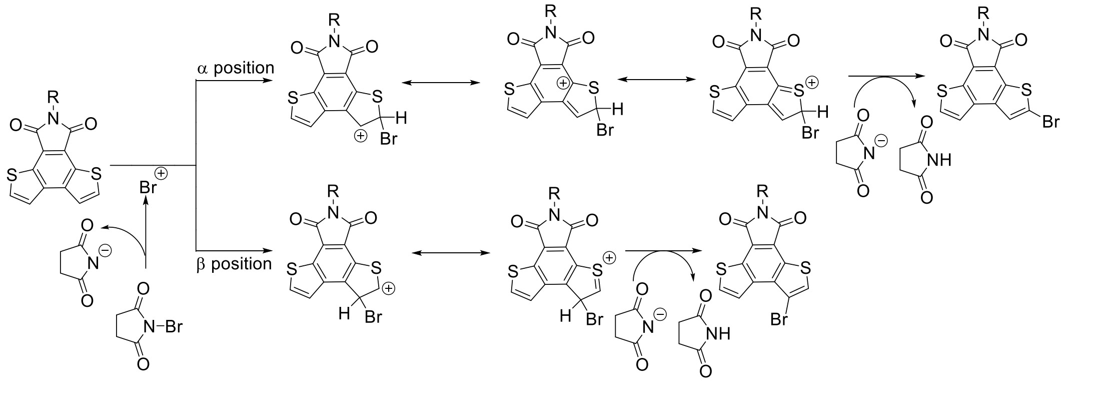

Scheme S2. Brominated reaction mechanism of **DTI** in the presence of NBS.[1-3]

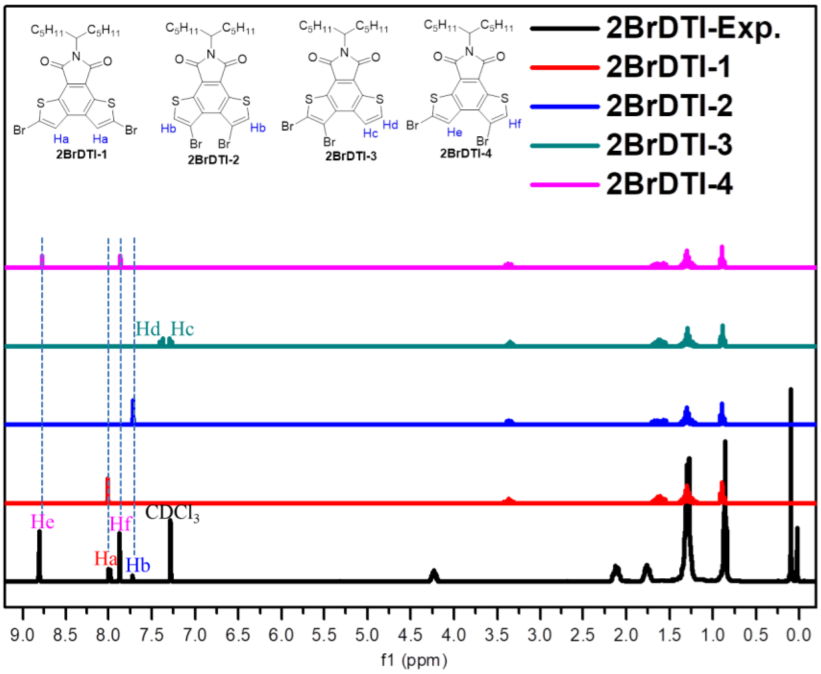

Figure S2. The experimental and theoretical ^{1}H NMR spectra of **2BrDTI**.

Table S1. Crystallographic data for **DTI2Mes** (Deposition Number 1983261).

Formula	Molecular weight (g mol^{-1})	Crystal system	Space group	Hall group	Completeness
C41H47NO2S2	649.92	Monoclinic	P1 21/n	-P 2yn	99.7%
a (Å)	b (Å)	c (Å)	α (°)	β (°)	γ (°)
18.046	10.5384	19.795	90.000	97.379	90.000
T (K)	θ_{max} (°)	F (000)	N_{ref}	Dx (g cm^{-3})	S (all)
300	28.273	1824.0	9206	1.603	2.779

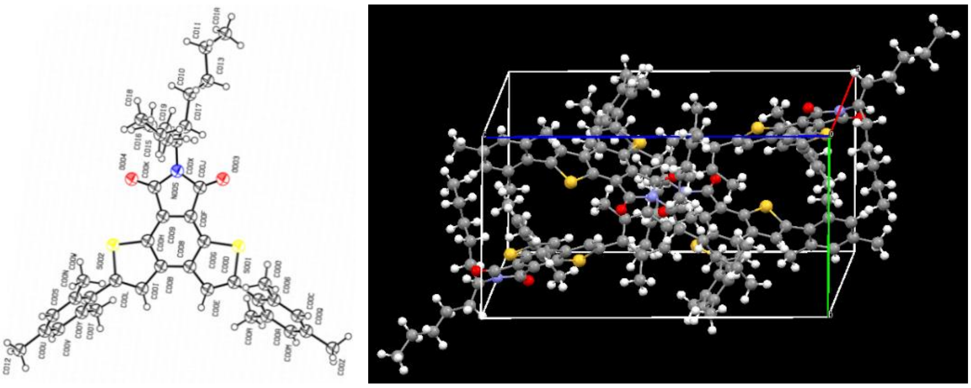

Figure S3. Oak ridge thermal ellipsoid plots (ORTEP) view of the molecular structures and packing structure of **DTI2Mes**.

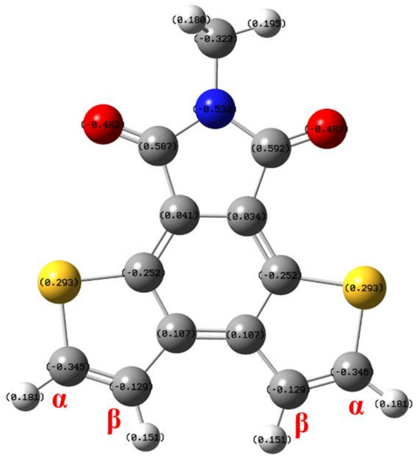

Figure S4. The Mulliken charge distribution of **DTI**. Structures and energies calculated with DFT B3LYP/6-31G level.

3. Characterization of DTI-based materials

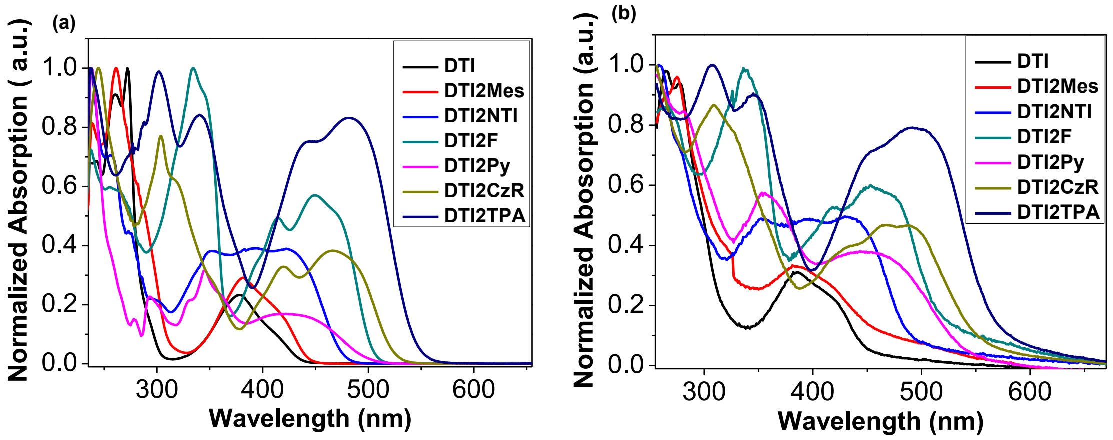

Figure S5. (a) UV-Vis absorption of **DTI**-based conjugated molecules in dilute DCM solutions (10^{-6} M) and (b) in the films spin-cast on quartz.

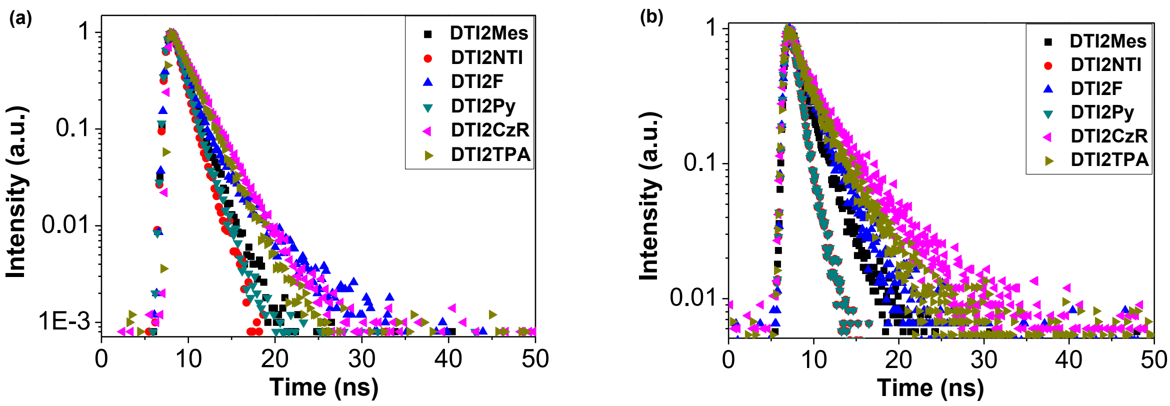

Figure S6. (a) Fluorescent lifetime tests of **DTI**-based conjugated molecules in dilute DCM solutions (10^{-6} M) and (b) in the spin-coated films.

Table S2. CV data for **DTI**-based conjugated molecules.

	E_{onset}^{red} (V)	E_{onset}^{ox} (V)	LUMO (eV)	HOMO (eV)	$LUMO_{cal}$ (eV)	$HOMO_{cal}$ (eV)	E_g^{cal} (eV)	E_g (eV)	E_g^{opt} (eV)
**DTI**	-1.28	1.49	-3.33	-6.10	-2.17	-6.09	3.92	2.77	2.78
**DTI2Mes**	-1.10	1.44	-3.51	-6.05	-2.42	-6.00	3.58	2.54	2.75
**DTI2NTI**	-1.09	1.39	-3.52	-6.00	-2.45	-5.88	3.43	2.48	2.53
**DTI2F**	-1.05	1.29	-3.56	-5.90	-2.49	-5.43	2.94	2.34	2.38
**DTI2Py**	-1.13	1.10	-3.48	-5.71	-2.41	-5.34	2.93	2.23	2.31
**DTI2CzR**	-1.15	0.70	-3.46	-5.31	-2.26	-5.13	2.87	1.85	2.24
**DTI2TPA**	-1.08	0.64	-3.53	-5.25	-2.41	-5.06	2.65	1.72	2.17

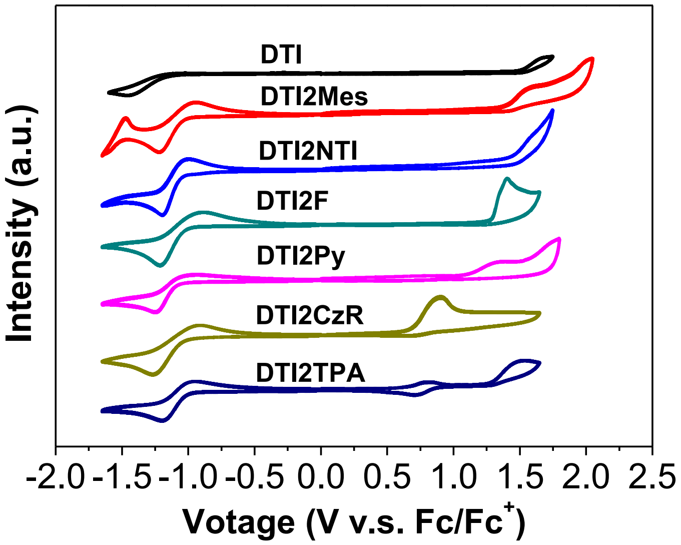

Figure S7. Cyclic voltammogram (CV) curves of **DTI**-based molecules.

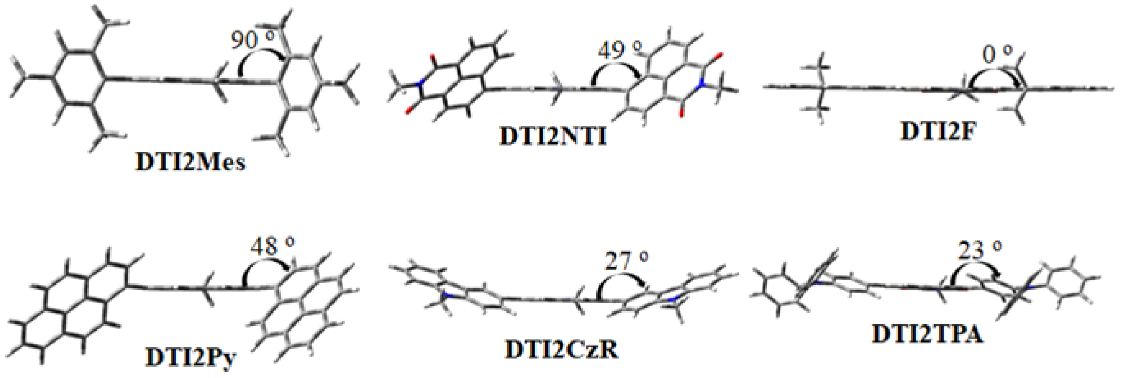

Figure S8. Side views of the optimized geometries obtained from DFT calculations at the B3LYP/6-31G level.

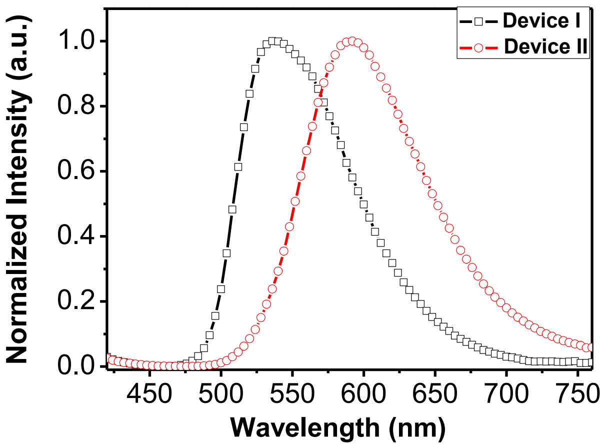

Figure S9. Electroluminescence (EL) spectra of the devices based on **DTI2F** and **DTI2CzR**.

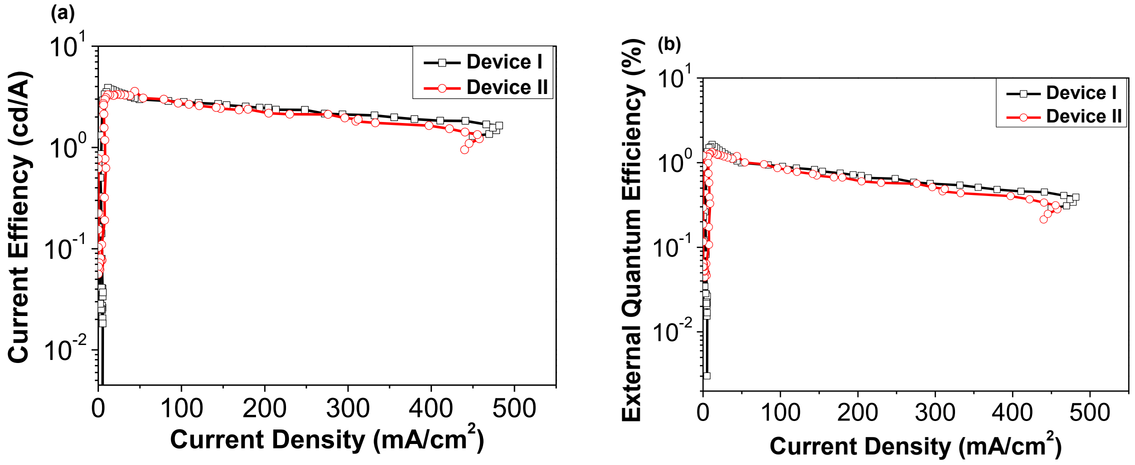

Figure S10. (a) Current efficiency-current density curves and (b) EQE-current density curves of the devices based on **DTI2F** and **DTI2CzR**.

4. MALDI-TOF and NMR Spectra

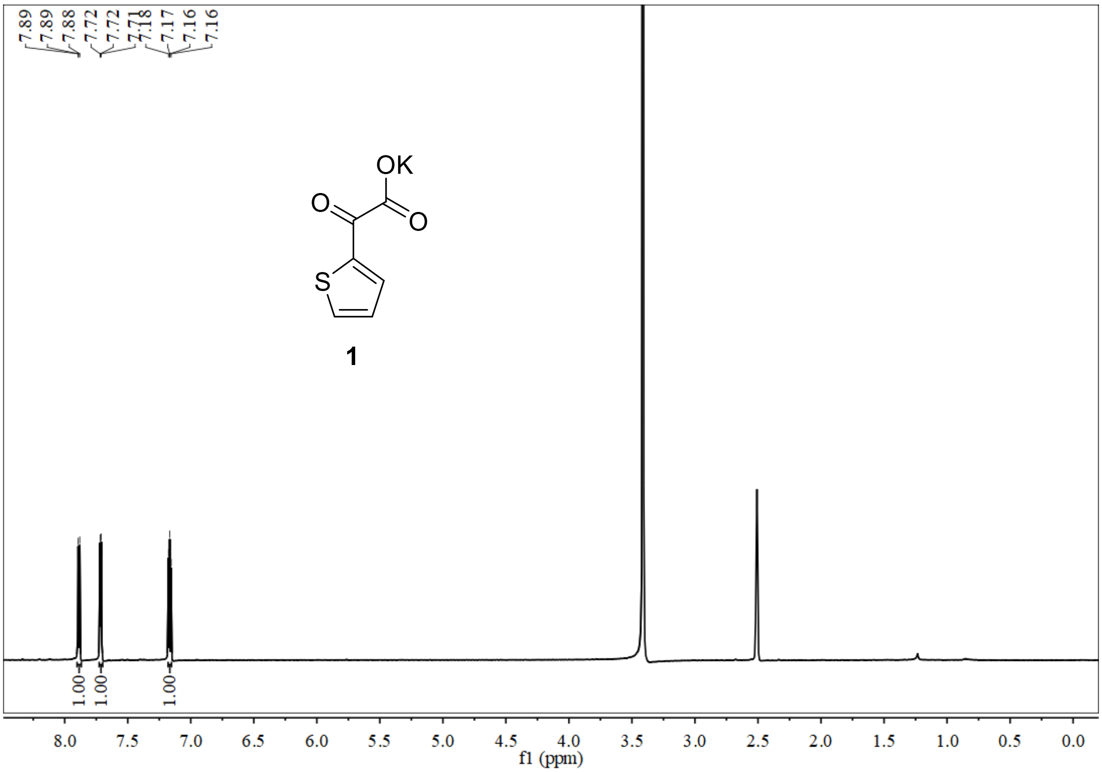

Figure S11. ^{1}H NMR of **1**.

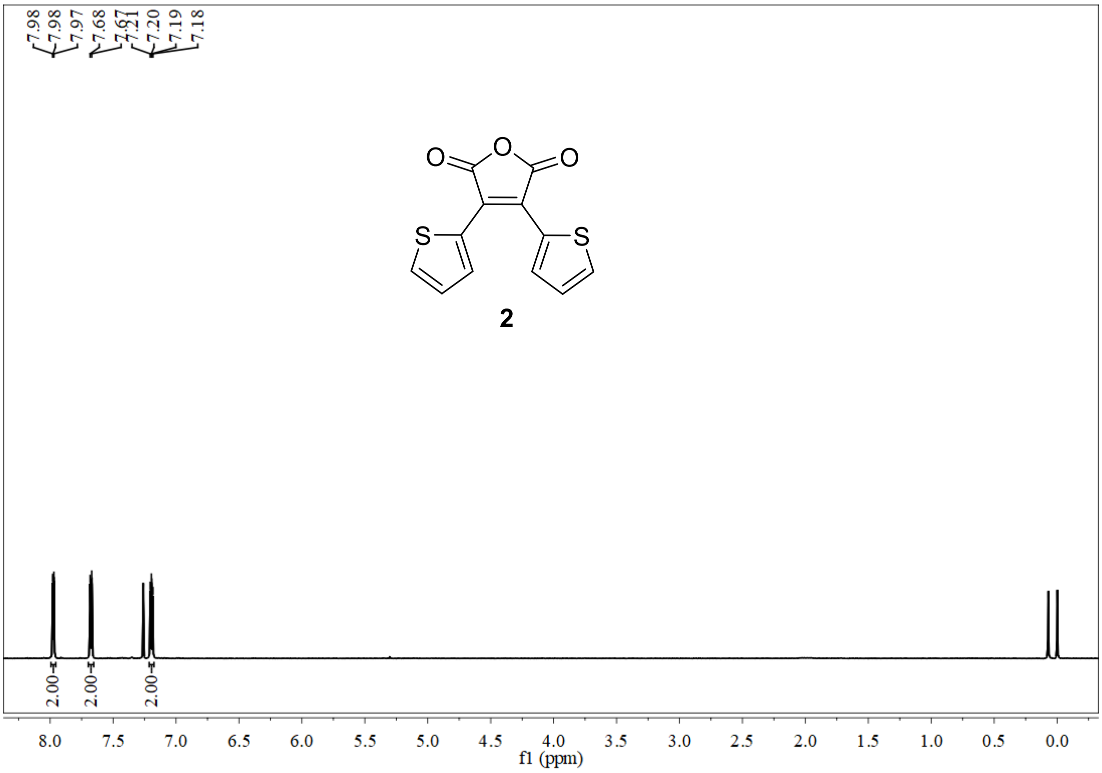

Figure S12. ^{1}H NMR of **2**.

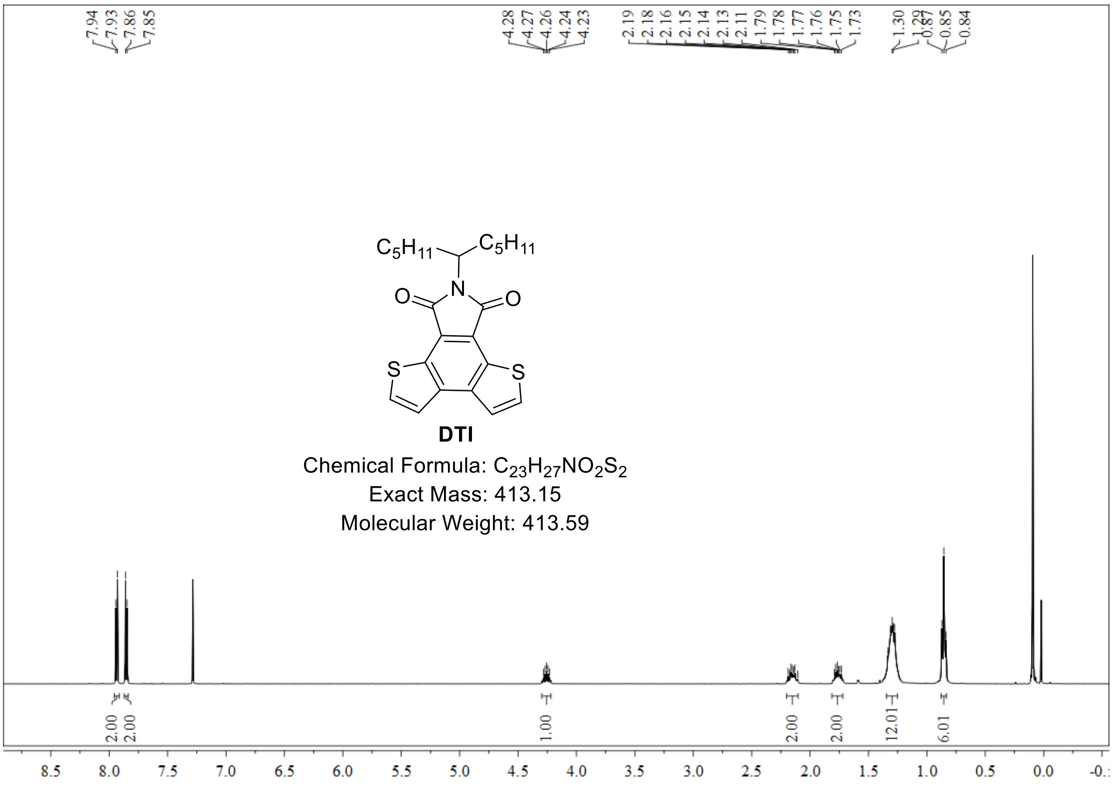

Figure S13. ^{1}H NMR of **DTI**.

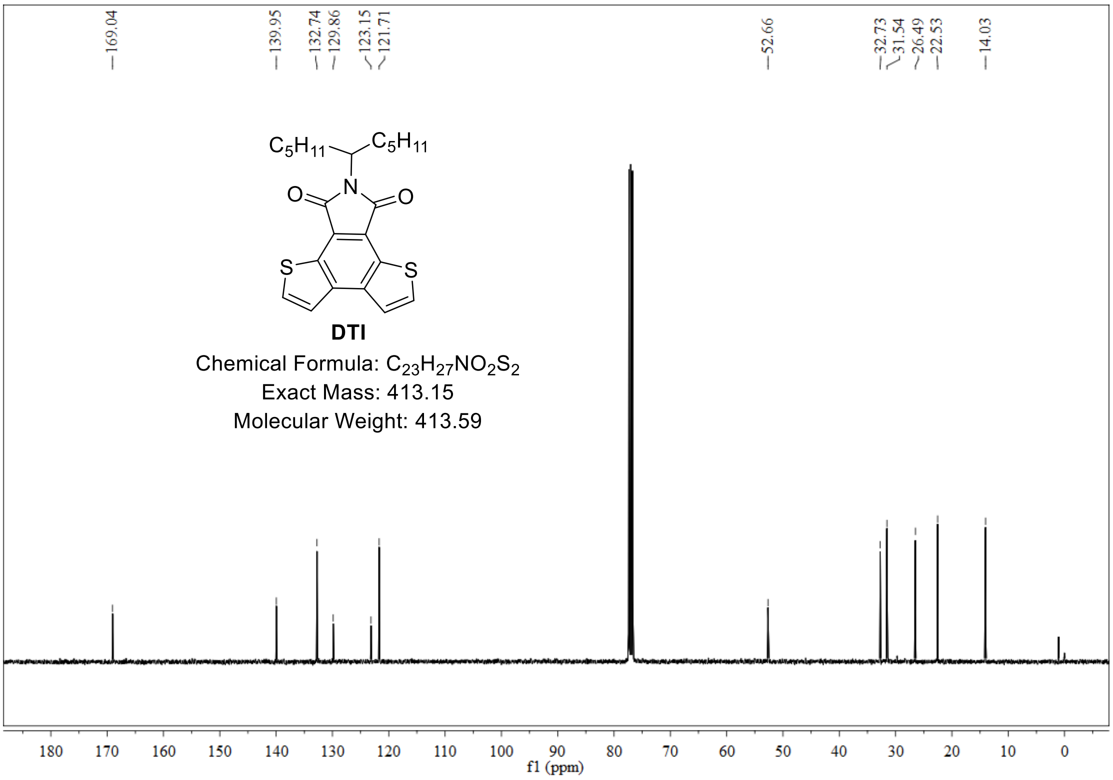

Figure S14. ^{13}C NMR of **DTI**.

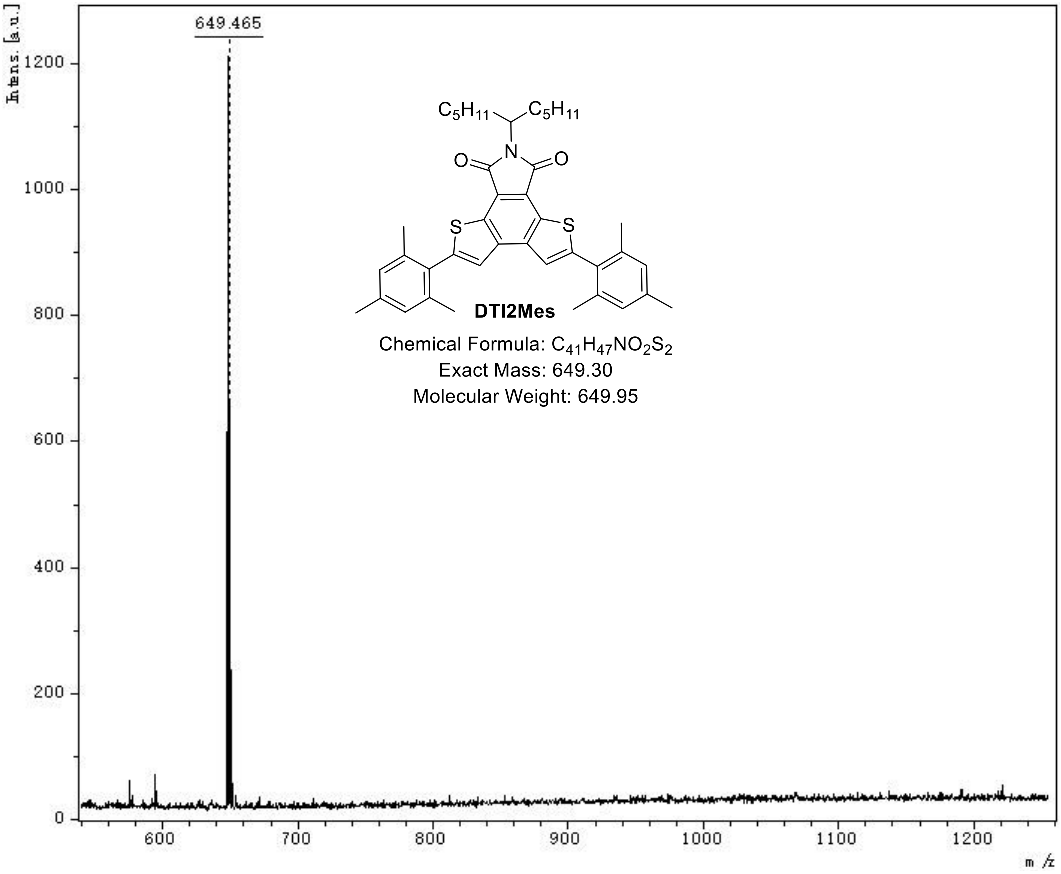

Figure S15. MALDI-TOF of **DTI2Mes**.

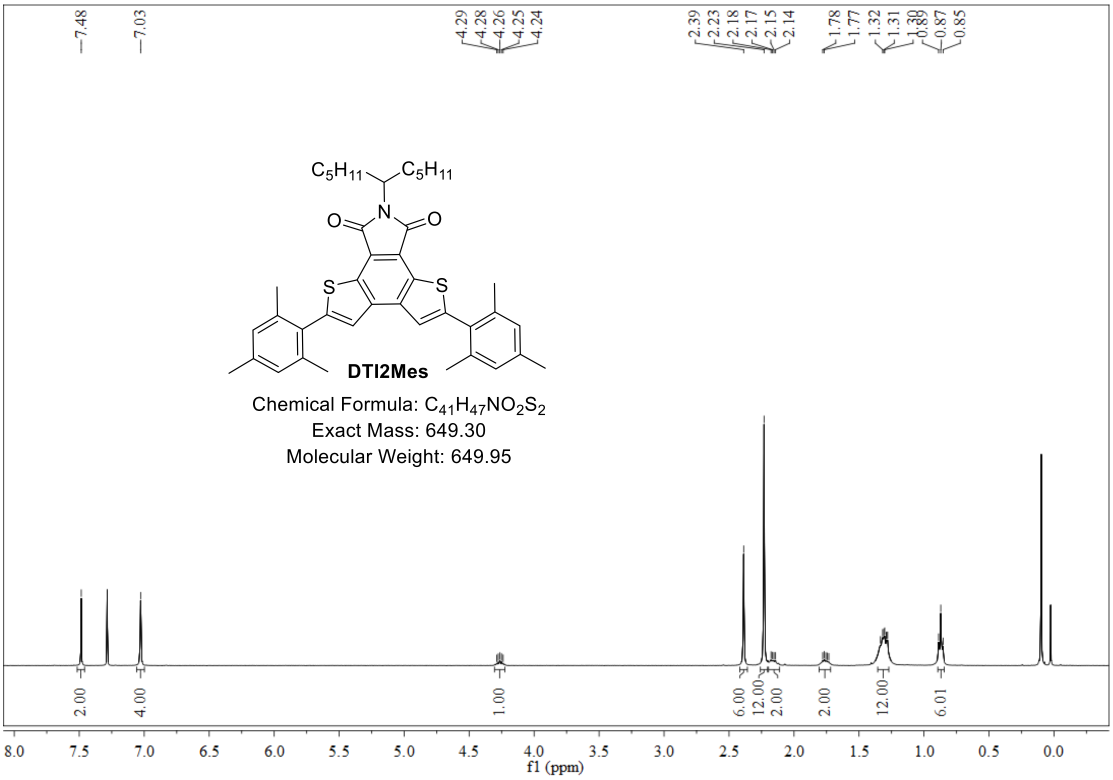

Figure S16. ^{1}H NMR of **DTI2Mes**.

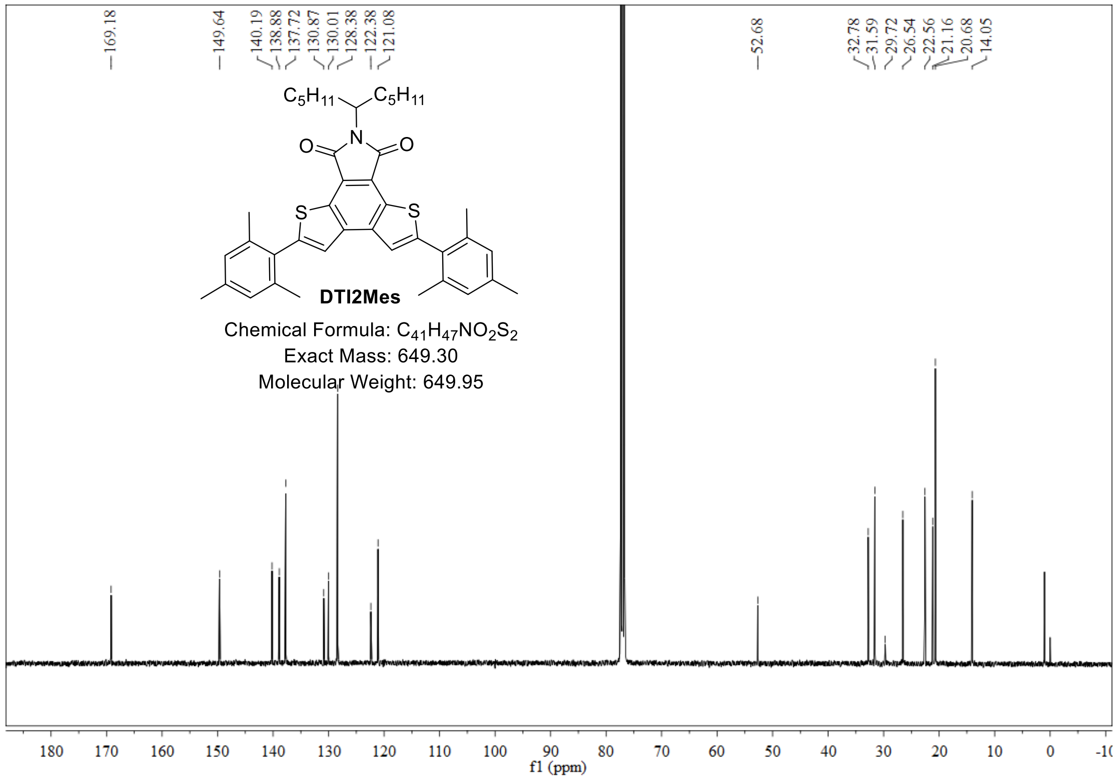

Figure S17. ^{13}C NMR of **DTI2Mes**.

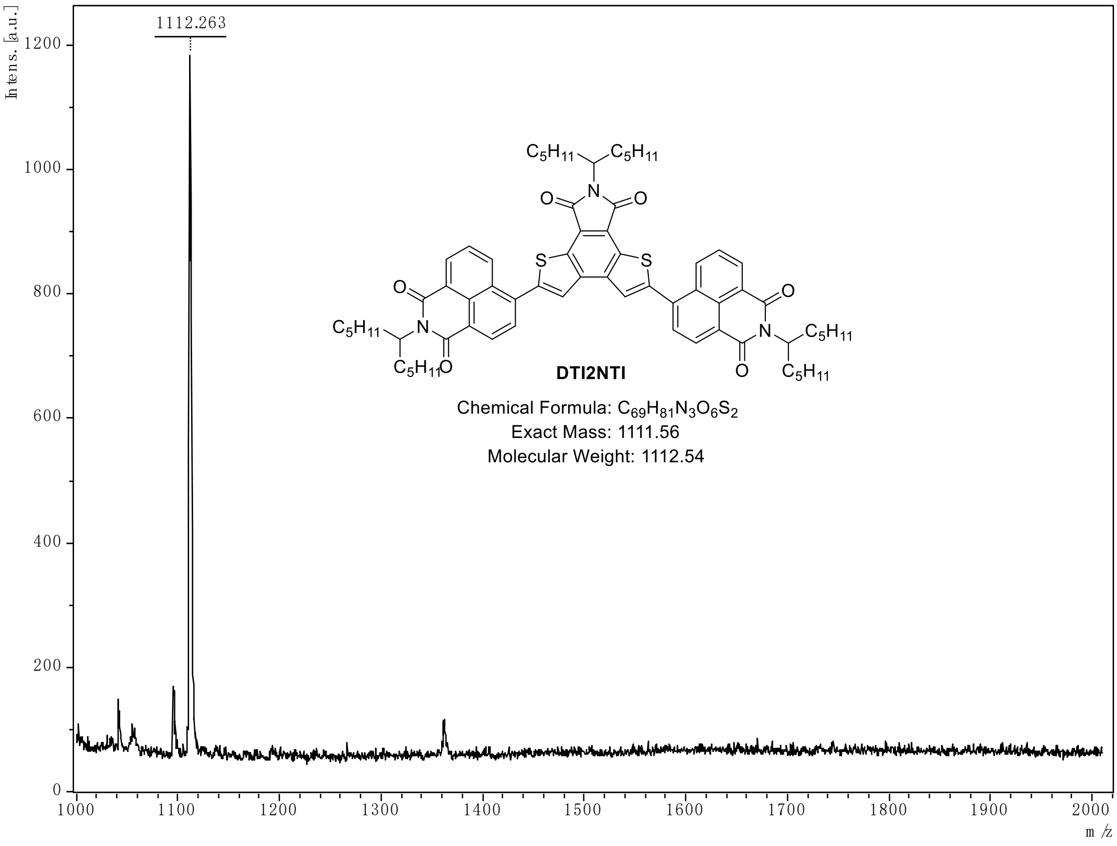

Figure S18. MALDI-TOF of **DTI2NTI**.

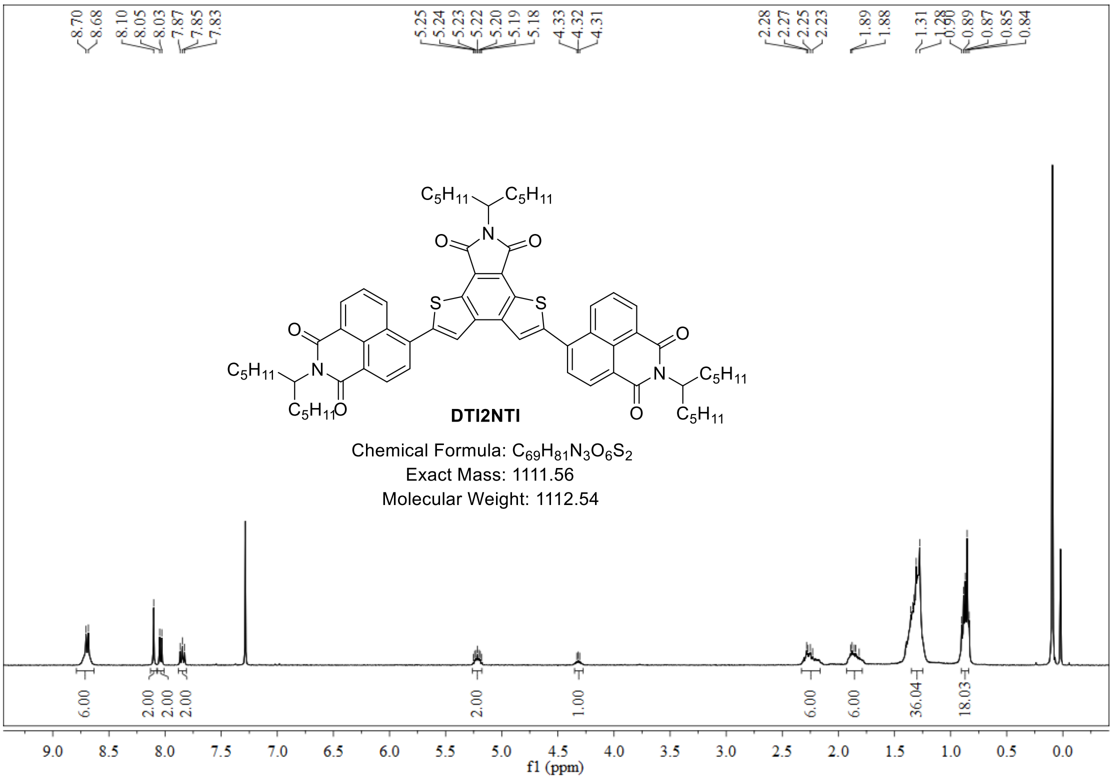

Figure S19. ^{1}H NMR of **DTI2NTI**.

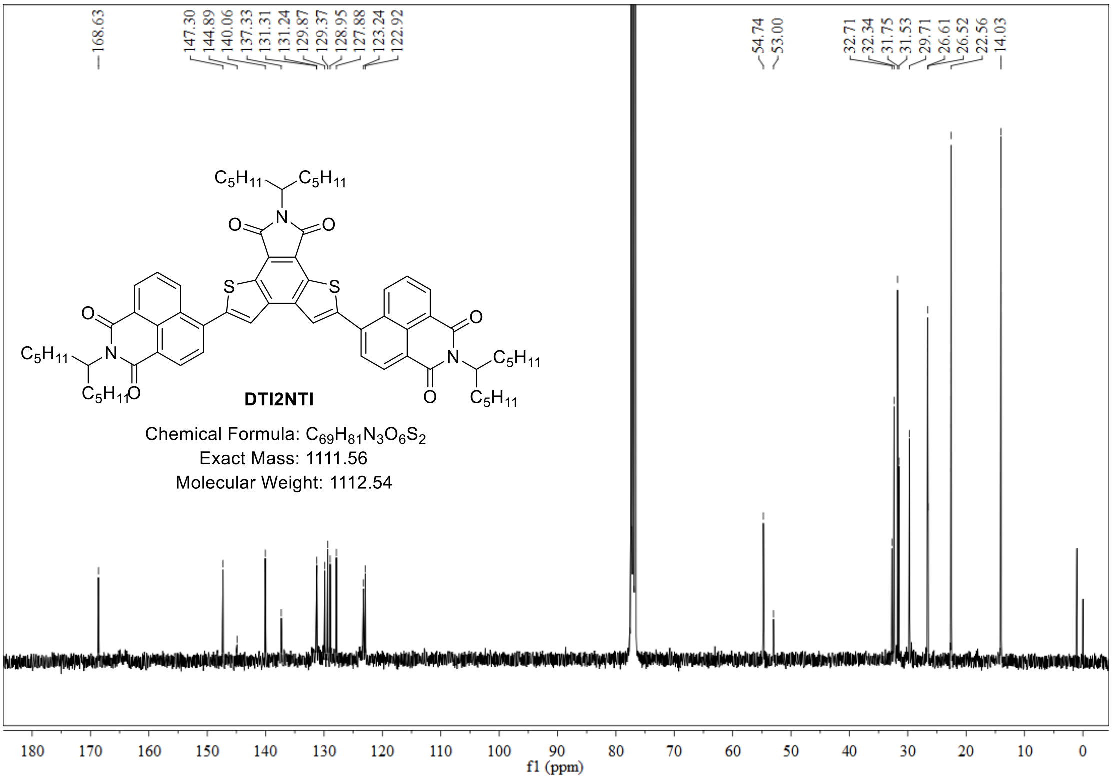

Figure S20. ^{13}C NMR of **DTI2NTI**.

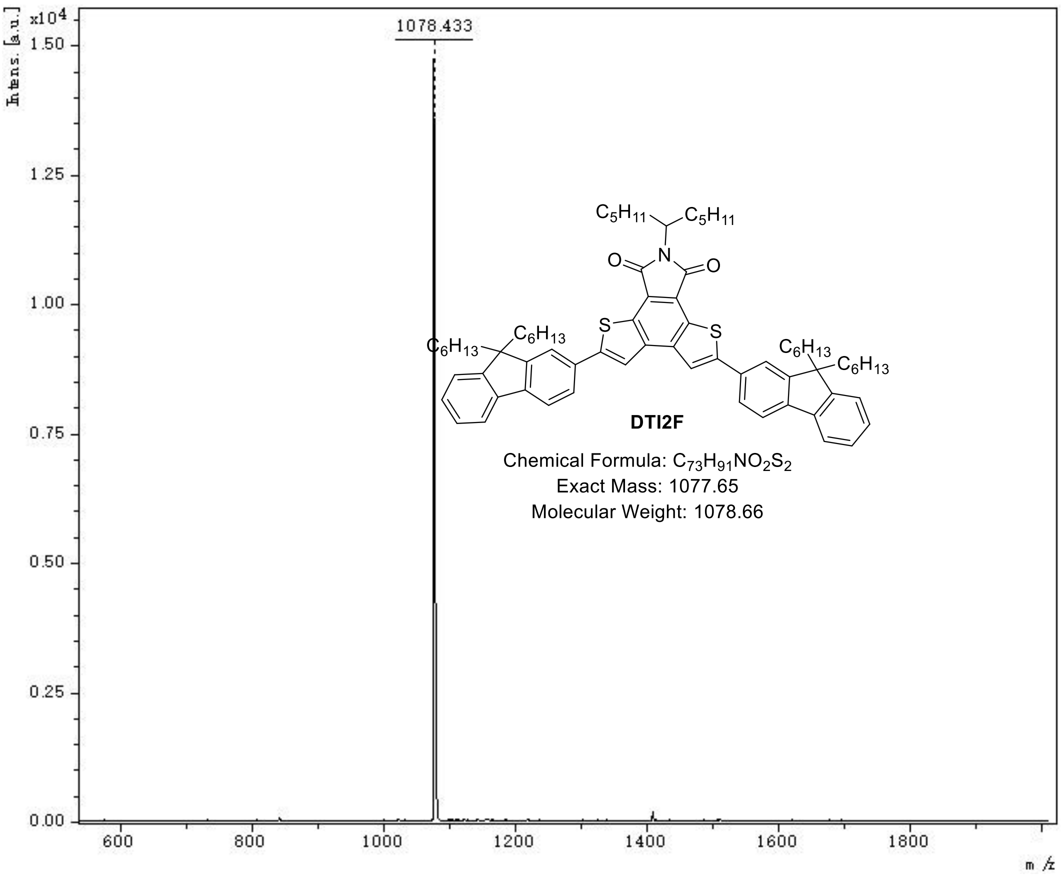

Figure S21. MALDI-TOF of **DTI2F**.

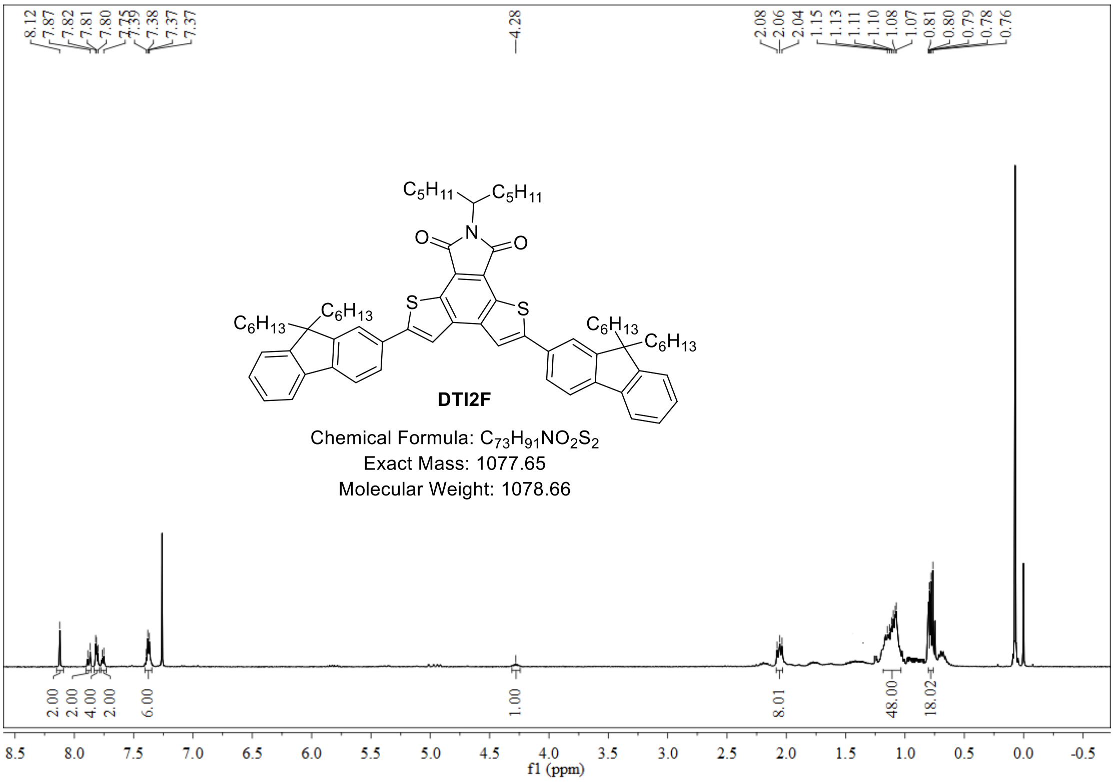

Figure S22. ^{1}H NMR of **DTI2F**.

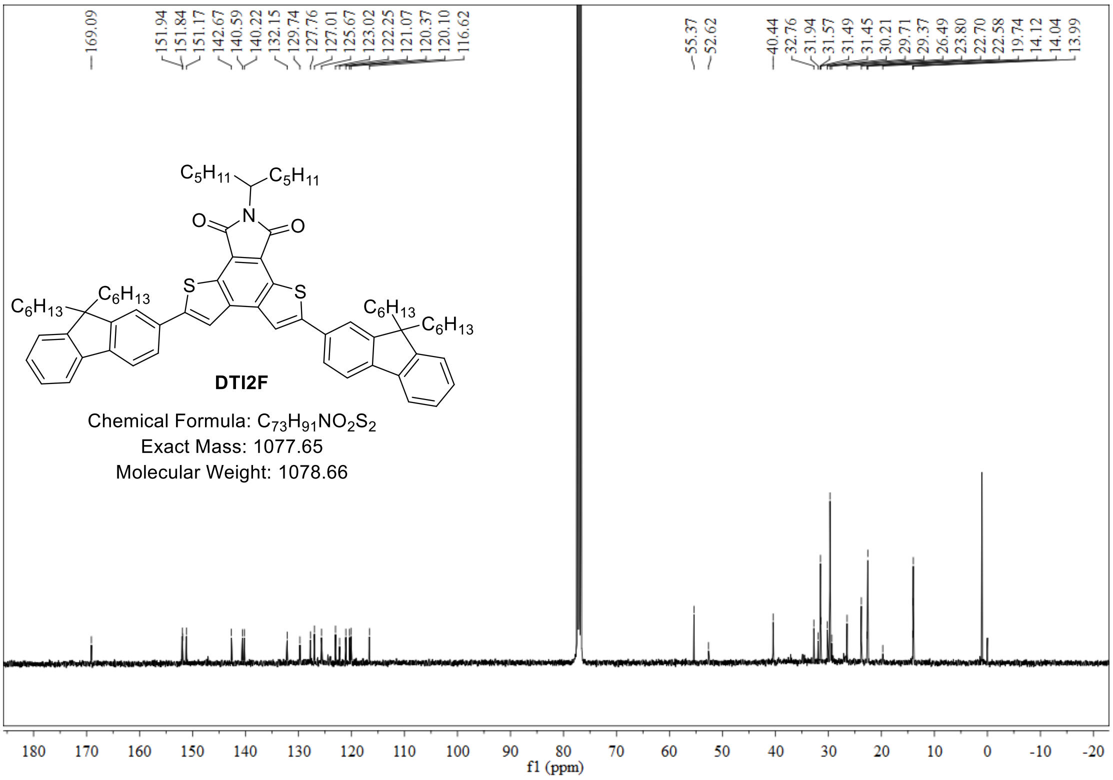

Figure S23. ^{13}C NMR of **DTI2F**.

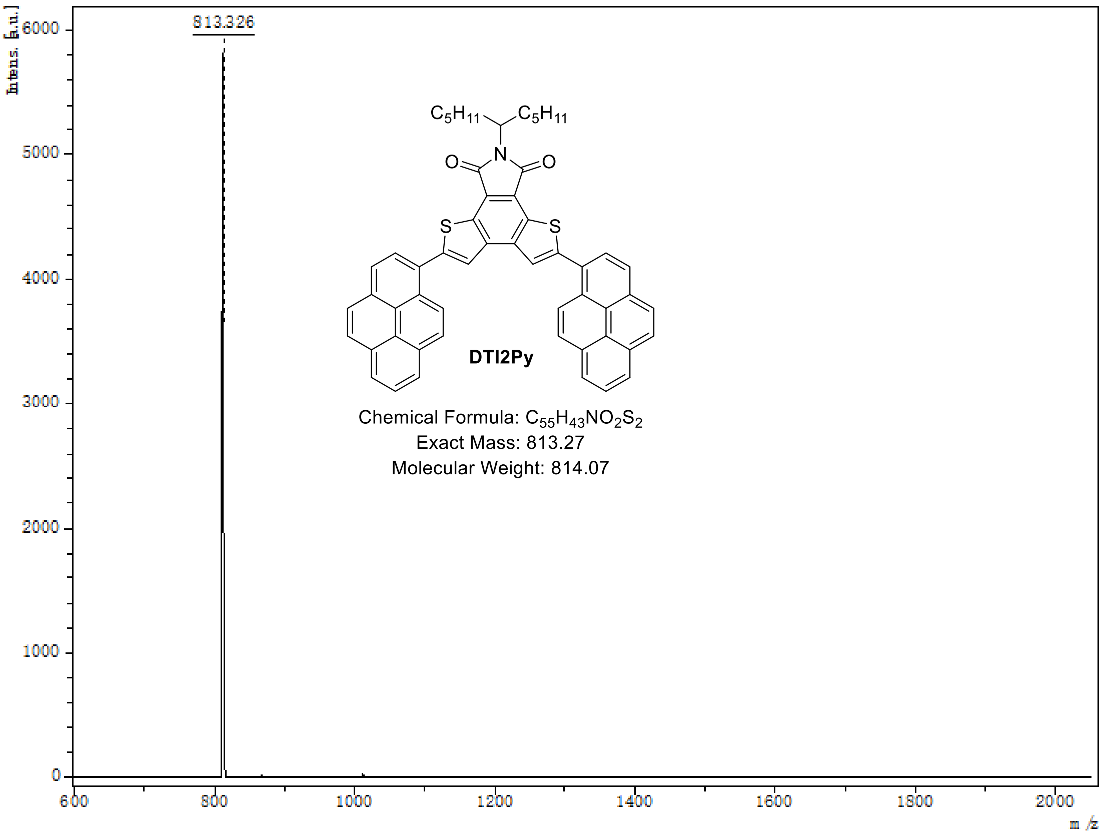

Figure S24. MALDI-TOF of **DTI2Py**.

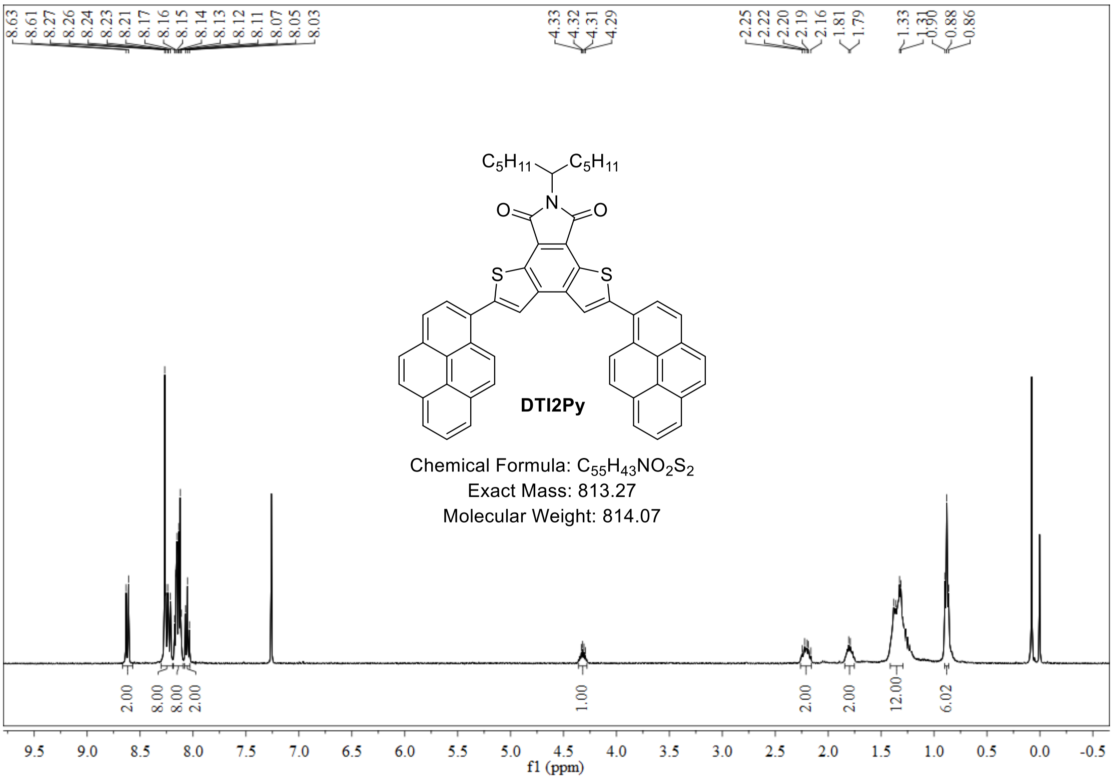

Figure S25. ^{1}H NMR of **DTI2Py**.

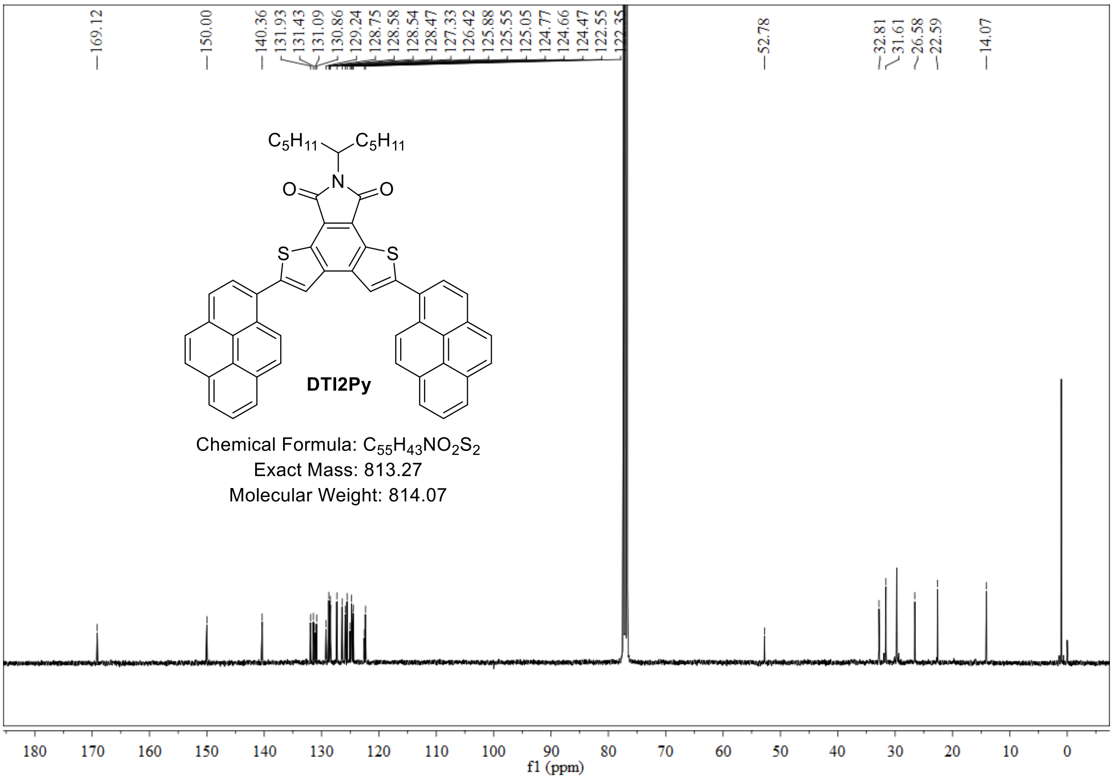

Figure S26. ^{13}C NMR of **DTI2Py**.

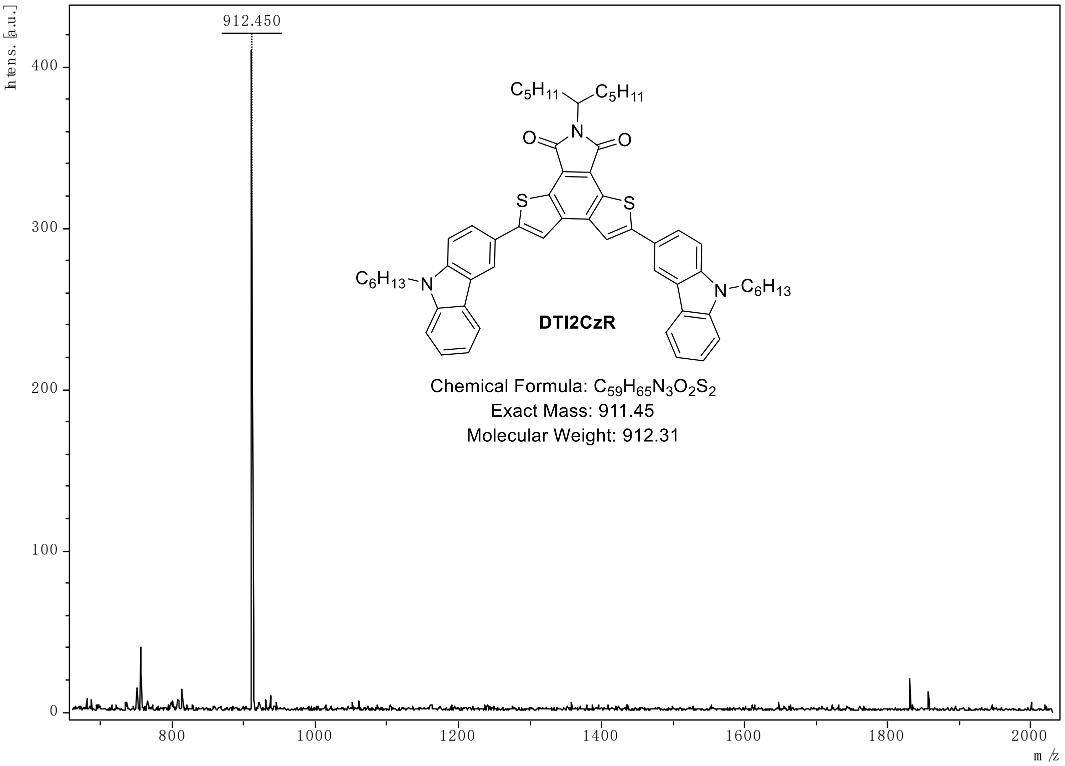

Figure S27. MALDI-TOF of **DTI2CzR**.

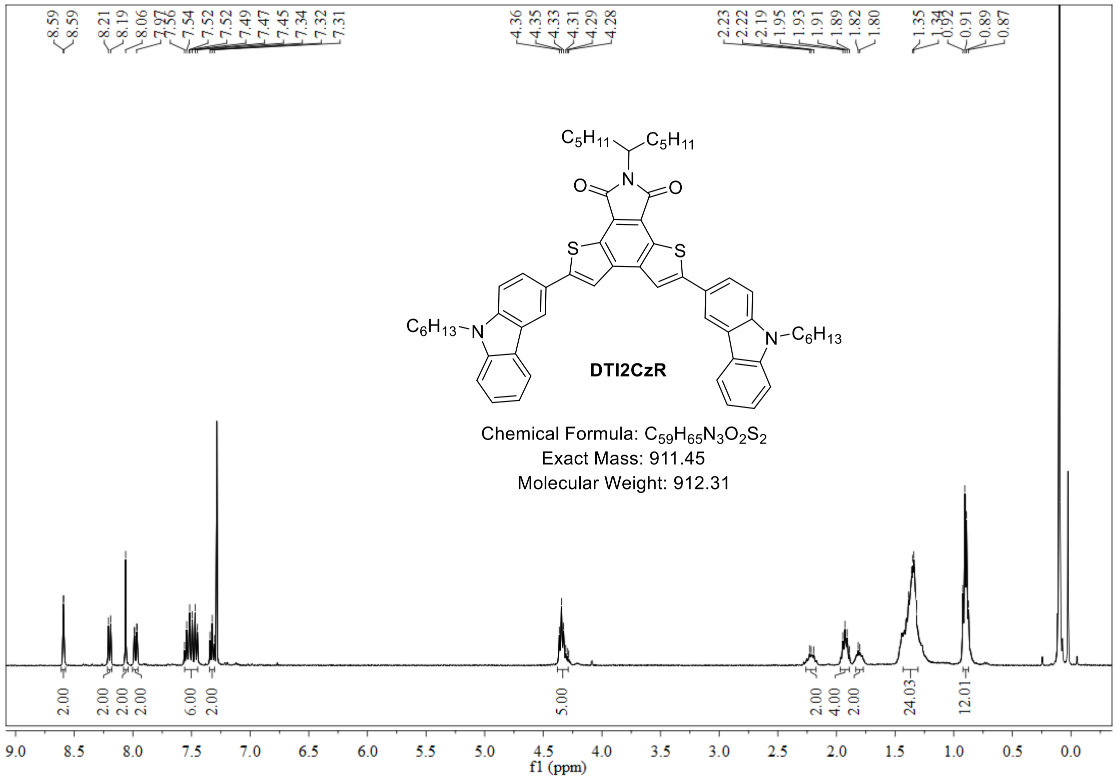

Figure S28. ^{1}H NMR of **DTI2CzR.**

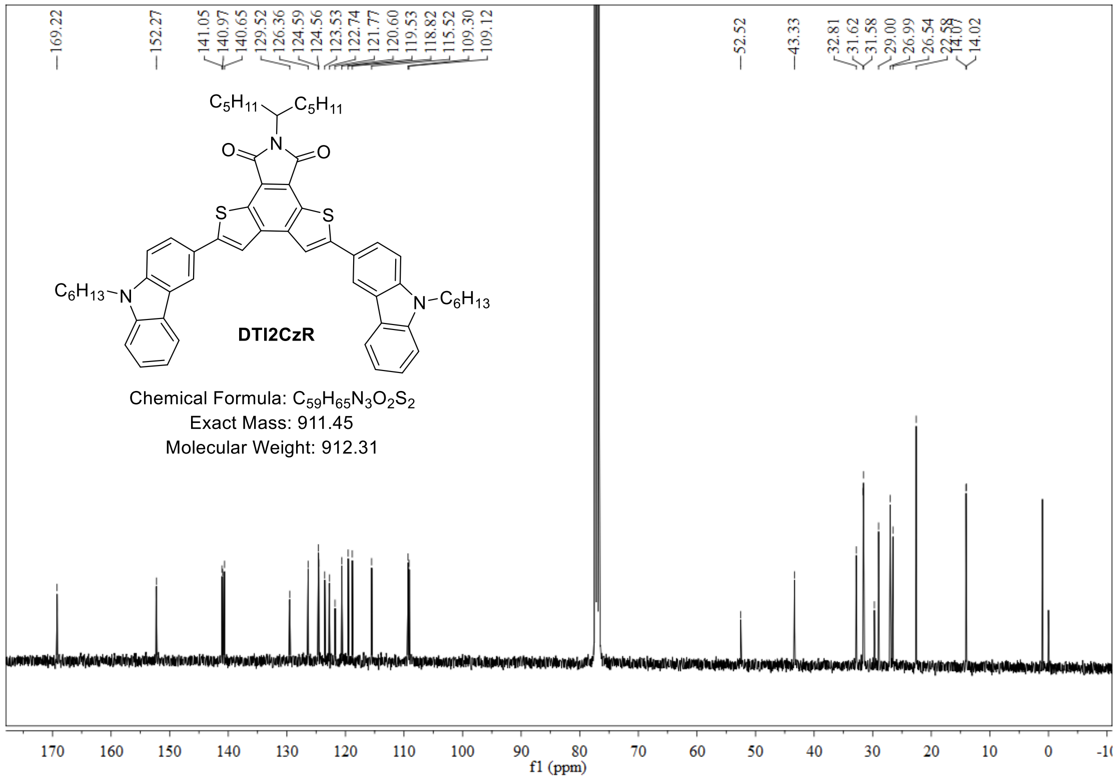

Figure S29. ^{13}C NMR of **DTI2CzR.**

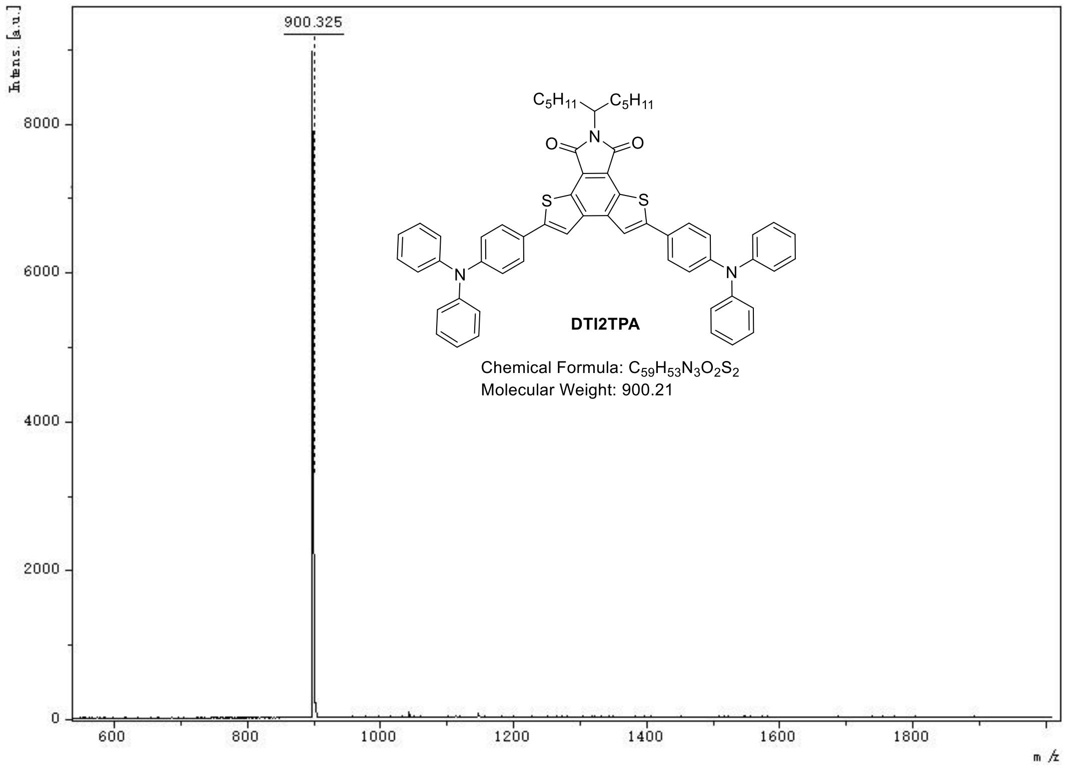

Figure S30. MALDI-TOF of **DTI2TPA.**

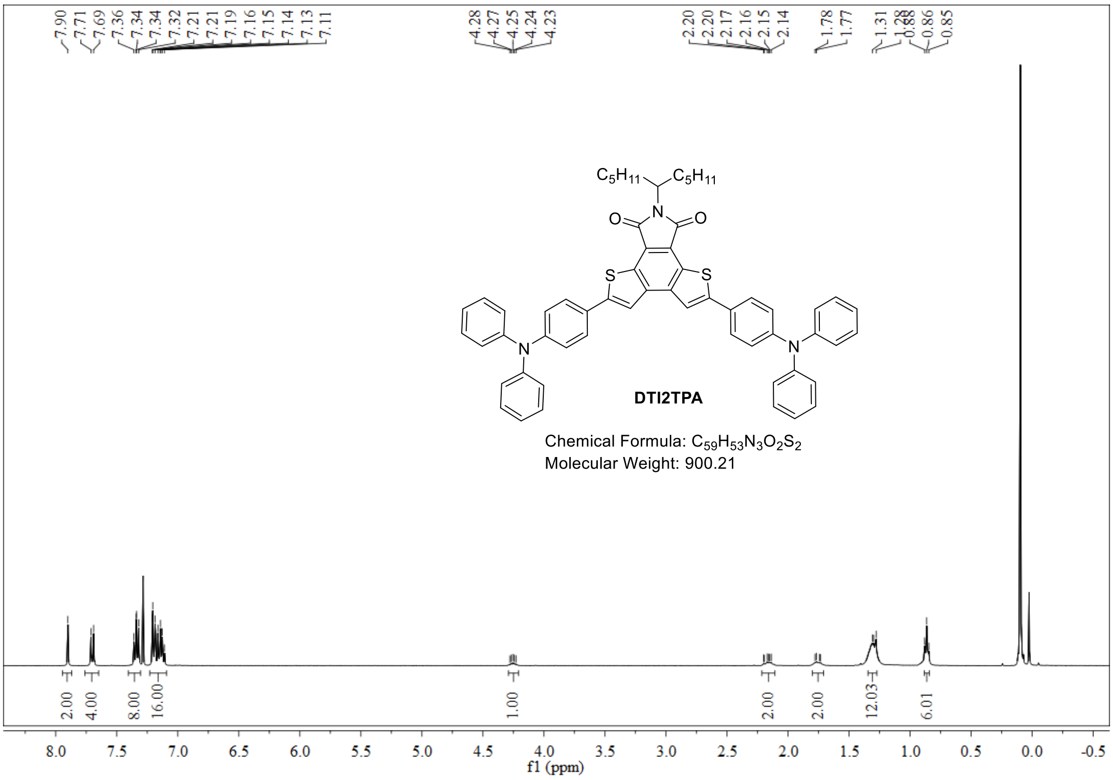

Figure S31. ^{1}H NMR of **DTI2TPA.**

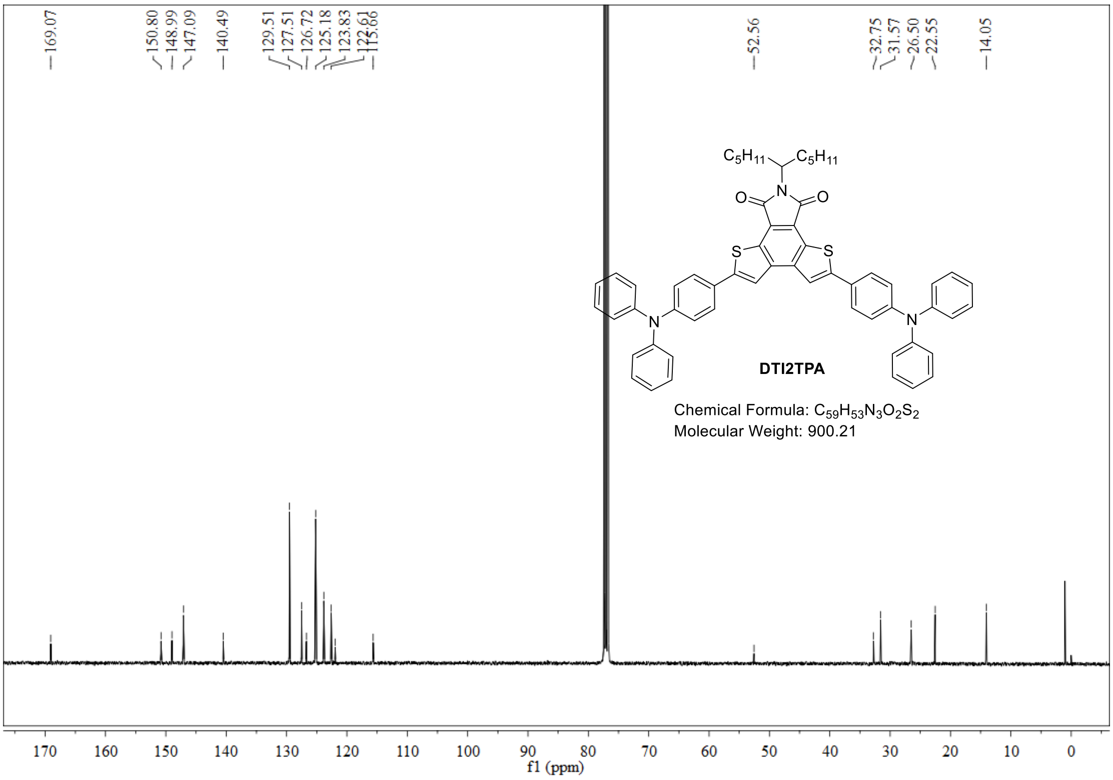

Figure S32. ^{13}C NMR of **DTI2TPA.**

6. References

[1] R. M. Kellogg, A. P. Schaap, E. T. Harper, and H. Wynbert, "Acid-catalyzed brominations, deuterations, rearrangements, and debrominations of thiophenes under mild conditions," *The Journal of Organic Chemistry*, vol. 33, no. 7, pp. 2902-2909, 1968.

[2] I. Saikia, A. J. Borah, and P. Phukan, "Use of bromine and bromo-organic compounds in organic synthesis," *Chemical Reviews*, vol. 116, no. 12, pp. 6837-7042, 2016.

[3] D. A. Guthrie and J. D. Tovar, "Conformation as a protecting group: A regioselective aromatic bromination en route to complex π-electron systems," *Organic Letters*, vol. 10, no. 19, pp. 4323-4326, 2008.
